# Overexpression of OSM and IL-6 impacts the polarization of pro-fibrotic macrophages and the development of bleomycin-induced lung fibrosis

**DOI:** 10.1038/s41598-017-13511-z

**Published:** 2017-10-16

**Authors:** Ehab A. Ayaub, Anisha Dubey, Jewel Imani, Fernando Botelho, Martin R. J. Kolb, Carl D. Richards, Kjetil Ask

**Affiliations:** 1 Department of Medicine, Firestone Institute for Respiratory Health, McMaster University and The Research Institute of St. Joe’s Hamilton, Hamilton, ON Canada; 2 Department of Pathology and Molecular Medicine, McMaster Immunology Research Centre, McMaster University, Hamilton, ON Canada

## Abstract

Although recent evidence indicates that gp130 cytokines, Oncostatin M (OSM) and IL-6 are involved in alternative programming of macrophages, their role in lung fibrogenesis is poorly understood. Here, we investigated the effect of transient adenoviral overexpression of OSM or IL-6 in mice during bleomycin-induced lung fibrosis. Lung fibrosis and M2-like macrophage accumulation were assessed by immunohistochemistry, western blotting, gene expression and flow cytometry. *Ex-vivo* isolated alveolar and bone marrow-derived macrophages were examined for M2-like programming and signalling. Airway physiology measurements at day 21 demonstrated that overexpression of OSM or IL-6 exacerbated bleomycin-induced lung elastance, consistent with histopathological assessment of extracellular matrix and myofibroblast accumulation. Flow cytometry analysis at day 7 showed increased numbers of M2-like macrophages in lungs of mice exposed to bleomycin and OSM or IL-6. These macrophages expressed the IL-6Rα, but were deficient for OSMRβ, suggesting that IL-6, but not OSM, may directly induce alternative macrophage activation. In conclusion, the gp130 cytokines IL-6 and OSM contribute to the accumulation of profibrotic macrophages and enhancement of bleomycin-induced lung fibrosis. This study suggests that therapeutic strategies targeting these cytokines or their receptors may be beneficial to prevent the accumulation of M2-like macrophages and the progression of fibrotic lung disease.

## Introduction

Interstitial fibrotic lung diseases are characterized by excessive tissue remodelling and accumulation of extracellular matrix (ECM)^1–6^, leading to a progressive loss of function. Although intensive research over the past few decades have led to increased understanding of the pathobiology of lung fibrosis and the first approvals of antifibrotic drugs^7^, the cellular and molecular mechanisms involved in the variable progression of fibrosis in patients diagnosed with lung fibrosis are still unclear. The accumulation of myofibroblasts that synthesize and deposit ECM is a hallmark feature of fibrotic lung disease^8–10^. Although it is poorly understood how myofibroblasts differentiate in the lung parenchyma, recent data suggest that alternatively activated macrophage (AAMs) may be critical in the myofibroblast transformation process^11–15^.

Gp130 cytokines, also referred to as the IL-6 family, are involved in various inflammatory and immunoregulatory mechanisms. They elicit their cellular effects by binding to specific receptor complexes that include the signal transducing molecule, glycoprotein 130 (gp130) and play a critical role in various cellular processes such as cell differentiation, proliferation, and hematopoiesis^16–19^. *In vivo*, hyper-activation of the gp130/STAT3 signalling pathway (following genetic manipulation) causes excessive pulmonary fibrosis in murine models and occurs independently of the canonical TGFβ/SMAD3 signalling pathway^20^. Concerning macrophage polarization, IL-6 has recently been shown to induce an alternatively activated phenotype in macrophages derived from adipose tissue by enhancing IL-4Rα expression and augmenting the IL-4 response^21^. Additionally, Komori *et al*. (2015) demonstrate that OSM facilitates M2 programming *in vitro* using adipose tissue-derived macrophages^22^. Moreover, OSM has previously been shown to induce IL-6 cytokine expression *in vivo*, following OSM overexpression in murine models^23^. Recently, cellular response to OSM was associated with enhanced IL-6 production and worse outcomes in inflammatory bowel disease^24^, providing a point of convergence to induce IL-6-mediated signalling and rationalizes the implication of these two cytokines in driving pathological wound repair processes. In this study, we utilized an adenovirus over-expression system to assess the effects of OSM and IL-6 on bleomycin-induced lung injury and fibrosis in C57BL/6 mice. Our results demonstrate that over-expression of OSM (AdOSM) or IL-6 (AdIL-6) *in vivo* is associated with alternative macrophage accumulation and an increased fibrogenic response to bleomycin. This data suggests that targeting both gp130 cytokines and alternative programming of macrophages may be a viable strategy to prevent lung fibrogenesis.

## Results

### Overexpression of OSM and IL-6 intensifies bleomycin-induced lung fibrosis

**Figure 1:**
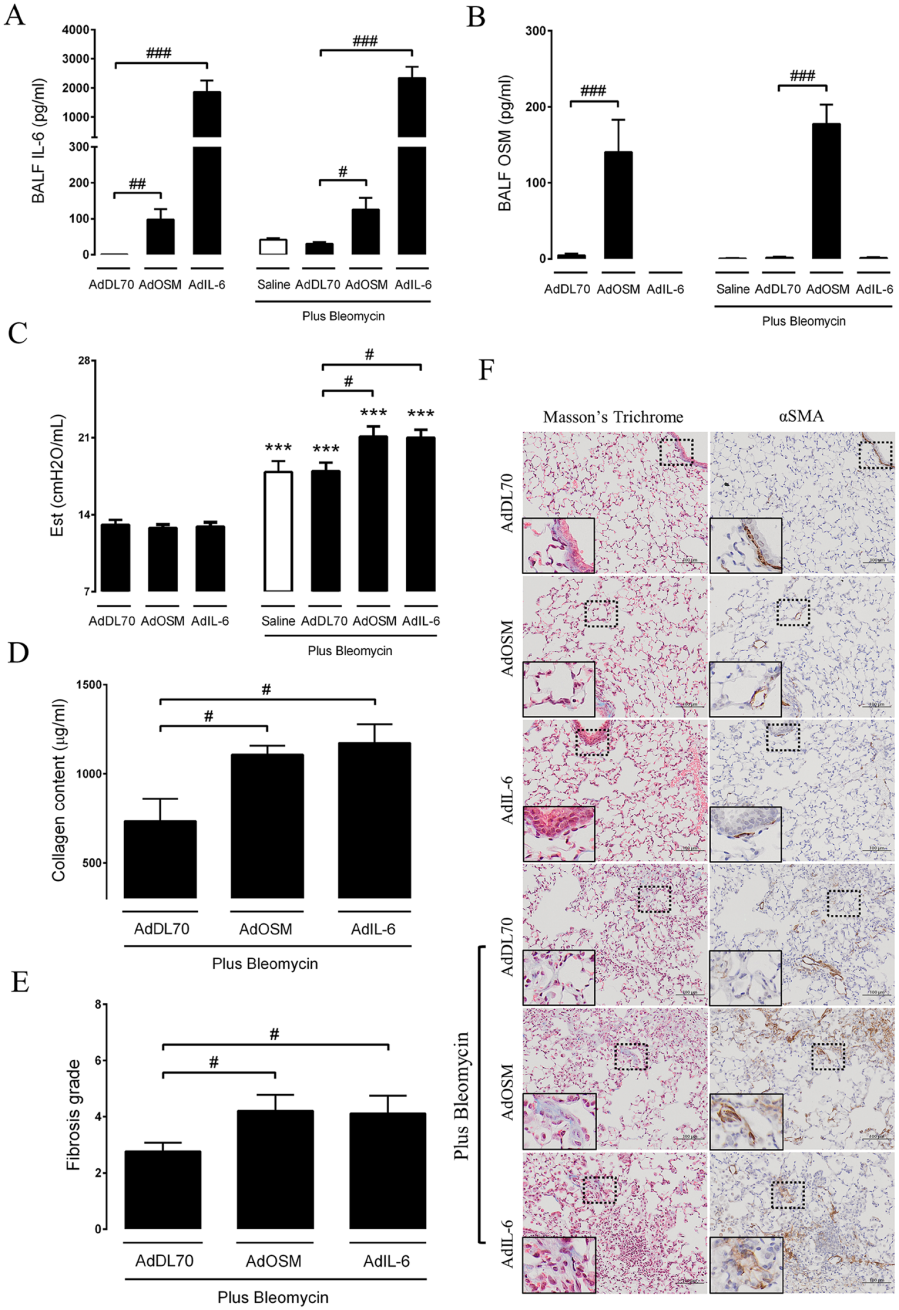
OSM and IL-6 worsen bleomycin-induced increase in lung elastance and bleomycin-induced fibrotic changes. Mice were intubated with AdDL70, AdOSM and AdIL-6, alone, or in combination with bleomycin (0.03 U/mouse). Additional control groups were included, receiving bleomycin plus saline with no adenovectors. Mice were sacrificed following 7 and 21 days of exposure, with the fibrotic outcome was assessed at day 21. (**A,B**) IL-6 and OSM were assessed in the BALF by ELISA (day 7). (**C**) Elastance derived from pressure-driven pressure volume loops are shown and graphed as an average value of all the animals per group. (**D**) Lung collagen content was assessed by Sircol collagen assay. (**E**) Ashcroft score demonstrating the grade of fibrosis and (**F**) representative images from Masson’s trichrome and αSMA stained lung sections. Bar graphs represent mean ± SEM from 5-8 mice per group. All samples were derived at the same time and processed in parallel. This figure shows one of two representative experiments. *P < 0.05; **P < 0.01; ***P < 0.001; ^#^P < 0.05; ^##^P < 0.01; ^###^P < 0.001; * represent a difference between bleomycin-exposed groups and their respective controls (Saline-Bleo vs. Saline, AdDL70- Bleo vs. AdDL70, AdOSM-Bleo vs. AdOSM, AdIL-6-Bleo vs. AdIL-6); ^#^represent a difference between the indicated groups. Significance was established using GraphPad, Prism 7.0 with one-way ANOVA using Newman-Keuls Multiple Comparison test.

To determine the effects of OSM and IL-6 on bleomycin-induced lung fibrosis, wild-type C57BL/6 mice were intratracheally administered adenovirus control vector or adenovirus encoding OSM or IL-6 to induce transient overexpression of OSM or IL-6, alone or in combination with bleomycin (0.03U/mouse). The BALF was collected after 7 and 21 days and assessed for either OSM or IL-6 by ELISA. As expected, we observed elevated levels of IL-6 (Fig. 1A) and OSM (Fig. 1B) in the BALF of mice treated with AdIL-6 or AdOSM, respectively, confirming consistent vector administration and cytokine overexpression. Mice treated with AdOSM alone or in combination with bleomycin showed elevated levels of IL-6 in the BALF, compared to AdDL70, confirming OSM induction of IL-6 as previously shown^23^. Both IL-6 and OSM levels were undetectable in BALF during the fibrotic phase (day 21) of the bleomycin-induced injury model (data not shown). After 21 days, the assessment of quasi-static lung elastance (Est) revealed that both AdOSM and AdIL-6 addition to bleomycin resulted in a significant increase in elastance as compared to control vectors (AdDL70) and to the adenoviral vectors AdOSM and AdIL-6 alone (Fig. 1C). As shown in Fig. 1C, the administration of bleomycin alone resulted in a similar response to bleomycin plus control vector (AdDL70). Therefore, all further experiments were conducted with AdDL70 as a control. The increase in lung stiffness was associated with increased collagen content (Fig. 1D) and Ashcroft fibrotic score based on Masson’s Trichrome staining **(**Fig. 1E **)**. Histological examination confirmed increased deposition of ECM (Masson’s Trichrome) and accumulation of alpha smooth muscle actin (αSMA) positive myofibroblasts in the lung parenchyma (Fig. 1F **)** of mice exposed to bleomycin and AdOSM and AdIL-6. By excluding large airways and blood vessels, digital analysis of αSMA confirmed enhanced parenchymal αSMA (Please see supplementary material, Fig. 1). Overall, these findings suggest that the overexpression of OSM and IL-6 results in an increased fibrotic response to bleomycin.

### Elevation of OSM and IL-6 lead to differential regulation of soluble mediators during bleomycin-induced lung injury

**Figure 2:**
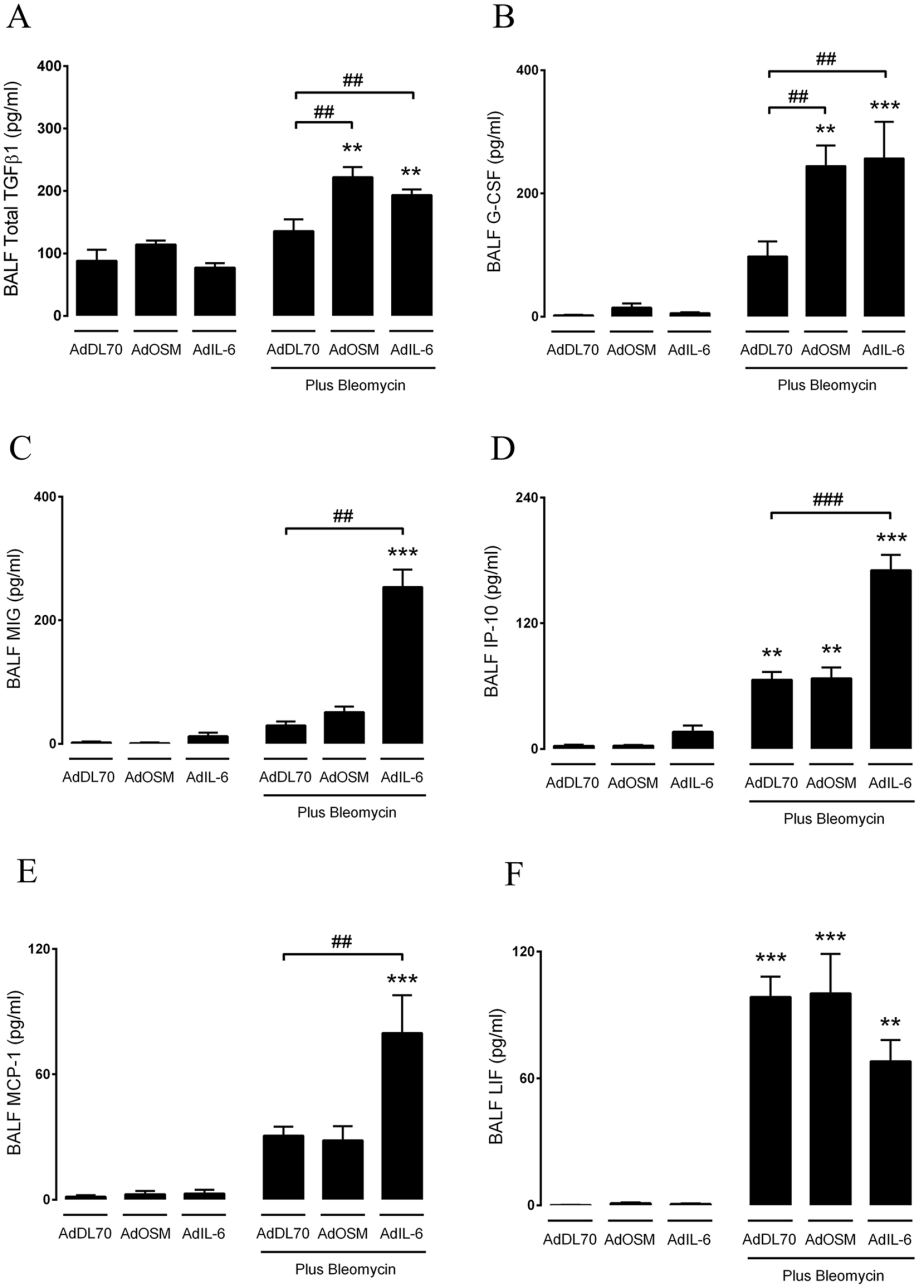
Enhanced production of several mediators in the lungs of bleomycin-exposed mice during IL-6 or OSM overexpression. Following intratracheal intubation of AdDL70, AdOSM and AdIL-6 alone, or in combination with bleomycin (0.03 U/mouse), mice were sacrificed after 7 days. Different cytokine/chemokine mediators were assessed in the BALF, either by ELISA or multiplex assay. The differentially regulated factors include (**A**) TGFβ1 (**B**) G-CSF (**C**) MIG (**D**) IP-10 (**E**) MCP-1 and (**F**) LIF. All other mediators were below the level of detection, and are therefore not included in this figure. Bar graphs represent mean ± SEM from 3-5 mice per group, ^*^P < 0.05; ^**^P < 0.01; ^***^P < 0.001; ^#^P < 0.05; ^##^P < 0.01; ^###^P < 0.001; ^*^represent a difference between bleomycin-exposed groups and their respective controls (AdDL70- Bleo vs. AdDL70, AdOSM-Bleo vs. AdOSM, AdIL-6-Bleo vs. AdIL-6); ^#^represent a difference between the indicated groups. Significance was established using GraphPad, Prism 7.0 with one-way ANOVA using Newman-Keuls Multiple Comparison test.

To investigate how OSM and IL-6 overexpression led to the increased fibrogenic response in bleomycin-induced fibrosis, we assessed pro-fibrotic and inflammatory mediators in the broncheoalveolar fluid (BALF) at day 7 after the administration of bleomycin and the adenovectors. Total TGFβ1 and G-CSF were increased in the lungs of mice exposed to both bleomycin plus AdOSM or AdIL-6 **(**Fig. 2A–B). In contrast, even though bleomycin caused a general increase in monokine-induced by gamma-interferon (MIG), interferon-gamma inducible-protein-10 (IP-10) and monocyte chemoattractant protein-1 (MCP-1/CCL2), mice receiving both bleomycin and AdIL-6 had an enhanced level of increase in these mediators (Fig. 2C–E), suggesting a selective capacity of IL-6 in their regulation. Even though leukemia inhibitory factor (LIF) was increased in response to bleomycin challenge, it was not further modulated by OSM or IL-6 overexpression **(**Fig. 2F **)**. All other mediators assessed were below the threshold limit of detection (not shown). Overall, these findings indicate that OSM and IL-6 over-expression leads to a differential induction of inflammatory chemokines during bleomycin-induced lung injury, suggesting that their individual fibrogenic properties might be different.

### OSM and IL-6 enhance the induction of markers associated with alternative programming of macrophages

**Figure 3:**
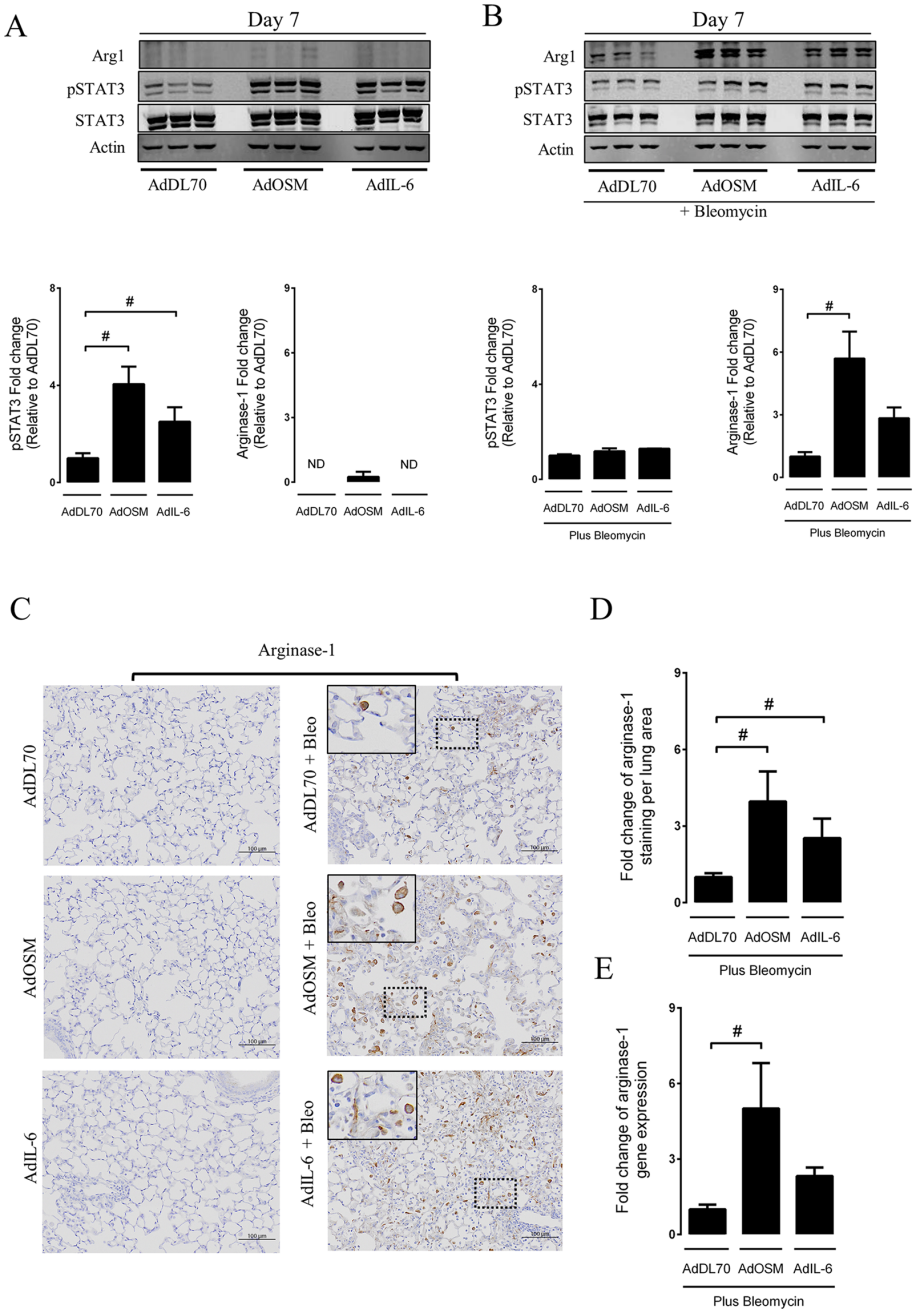
Increased induction of arginase-1 in the lungs of bleomycin-exposed groups during IL-6 or OSM overexpression. Following intratracheal intubation of AdDL70, AdOSM and AdIL-6, alone, or in combination with bleomycin (0.03 U/mouse), mice were sacrificed after 7 and 21 days. (**A**,**B**) Immunoblot images and densitometry analyses of STAT3, pSTAT3 and arginase-1 of lung homogenates following 7 days of exposure. (**C**–**E**) Representative arginase-1 immunostaining and quantification as well as arginase-1 mRNA signal in lung tissues following 7 days of exposure. Bar graphs represent mean ± SEM from 3–6 mice per group. All samples were derived at the same time and processed in parallel. The appropriate Western blot area depicting the antibody band was cropped and enclosed by black boxes, as indicated above. *P < 0.05; **P < 0.01; ***P < 0.001; ^#^P < 0.05; ^##^P < 0.01; ^###^P < 0.001; *represent a difference between bleomycin-exposed groups and their respective controls (AdDL70- Bleo vs. AdDL70, AdOSM-Bleo vs. AdOSM, AdIL-6-Bleo vs. AdIL-6); ^#^Represent a difference between the indicated groups. Significance was established using GraphPad, Prism 7.0 with one-way ANOVA using Newman-Keuls Multiple Comparison test.

As both IL-6 and OSM have been reported to influence alternative programming of macrophages, we next examined the presence of AAM in lungs of mice exposed to bleomycin plus OSM or IL-6 adenoviral vectors. As expected, 7 days after administration, at the peak of adenoviral expression, increased levels of phospho-STAT3 were observed in total lung homogenates **(**Fig. 3A **)**, confirming activation of the STAT3 pathway by overexpressed IL-6 or OSM in absence of bleomycin. This activation was not associated with a marked increase in arginase-1 expression **(**Fig. 3A **)**, as opposed to lungs exposed to bleomycin plus AdOSM or AdIL-6 **(**Fig. 3B **)**. Interestingly, the overall levels of pSTAT3 were not maintained in total lung homogenates exposed to both bleomycin and adenoviral control vectors, suggesting that the activation of the STAT3 pathway was not altered at this time-point **(**Fig. 3B **)**. For full-length pictures of western blots, please see supplementary Figure 6–9. To specifically examine the presence of AAMs in this model we proceeded with an immunohistochemical analysis of lungs exposed to adenoviral vectors alone or in combination with bleomycin at the same time-point. As observed on western blots in Fig. 3A, arginase-1 positive cells were not present in lungs exposed to the control vector or by AdOSM and AdIL-6 expression alone **(**Fig. 3C **)**. The specificity of the arginase-1 antibody was confirmed using IgG (negative control) and positive controls (see supplementary material, Fig. 2). The addition of bleomycin resulted in the appearance of multiple arginase-1 positive macrophage-like cells **(**Fig. 3C **)**. The quantification of the percentage of arginase-1 positive stain **(**Fig. 3D **)** in left lungs showed virtually identical outcome as the western blot observation noted above. These findings were also consistent with mRNA assessment of Arginase 1 from the corresponding right lungs (Fig. 3E), confirming the presence of alternative programming of macrophages in lungs exposed to bleomycin plus AdOSM or AdIL-6. We then examined arginase expression on macrophages isolated from lungs by flow cytometry.

### Increased accumulation of CD206+Arg1+macrophages in response to OSM and IL-6 during bleomycin-induced lung injury

**Figure 4:**
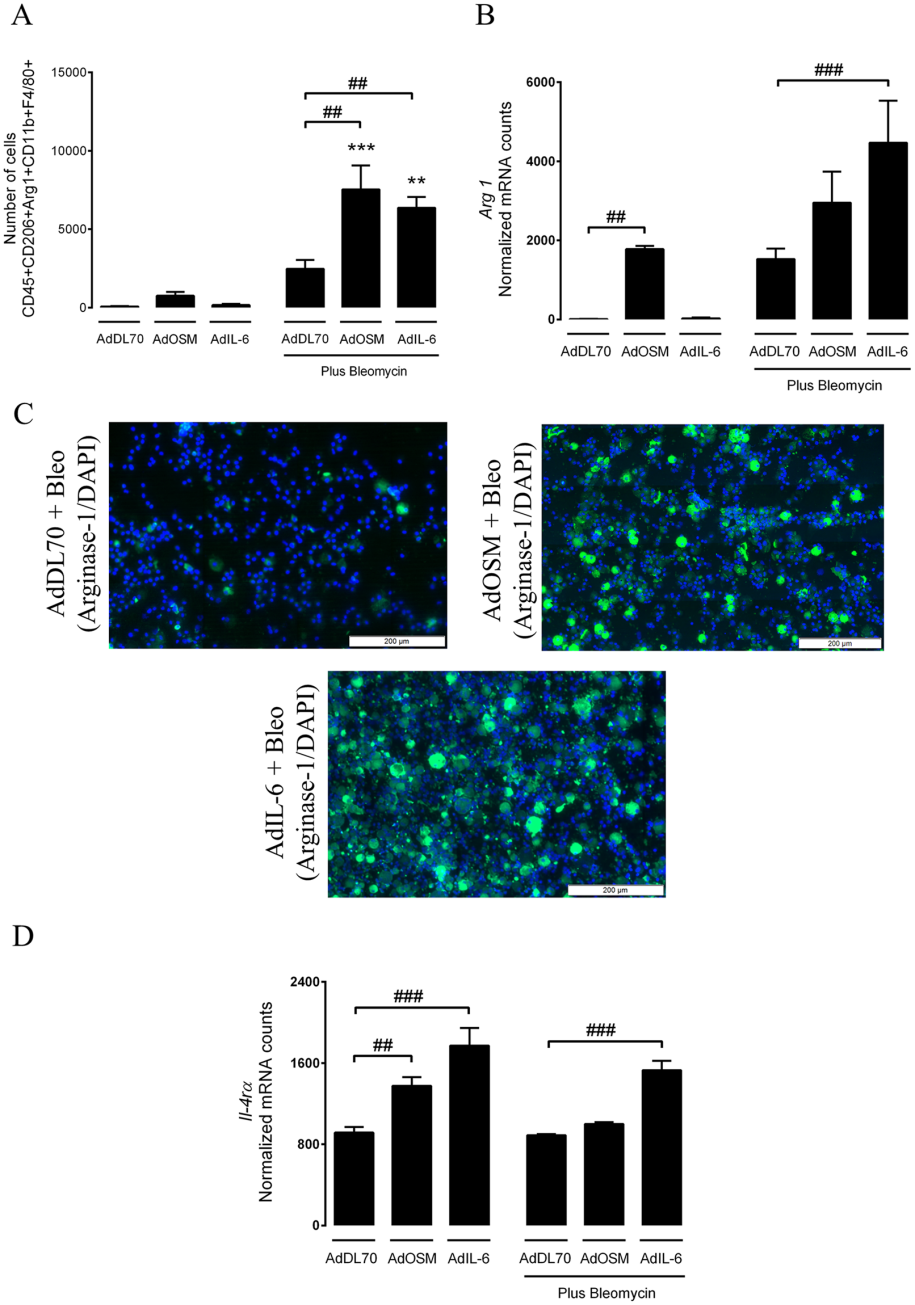
Increased accumulation of CD206+Arg1+ macrophages in the lungs of bleomycin-exposed mice during IL-6 or OSM overexpression. Following intratracheal intubation of AdDL70, AdOSM and AdIL-6, alone, or in combination with bleomycin (0.03 U/mouse) mice were sacrificed after 7 days. Lungs were processed for flow cytometry and AMs derived from BALF cells were isolated by adhesion and subsequently subjected to RNA analysis and Nanostring gene expression. (**A**) A graph showing absolute numbers of CD45+ CD206+ CD11b+Arg1+ F4/80+ cells. (**B,C**) graphs showing *Arg1* mRNA expression of adhered AMs and arginase-1/DAPI immunofluorescence staining of BALF cells. (**D**) *Il-4rα* mRNA expression of adhered AMs. All samples were derived at the same time and processed in parallel. Bar graphs represent mean ± SEM from 3–5 mice per group, *P < 0.05; **P < 0.01; ***P < 0.001; ^#^P < 0.05; ^##^P < 0.01; ^###^P < 0.001; *represent a difference between bleomycin-exposed groups and their respective controls (AdDL70- Bleo vs. AdDL70, AdOSM-Bleo vs. AdOSM, AdIL-6-Bleo vs. AdIL-6); ^#^represent a difference between the indicated groups. Significance was established using GraphPad, Prism 7.0 with one-way ANOVA using Newman-Keuls Multiple Comparison test.

The expression of CD206 and arginase-1 were assessed on lung-derived macrophages by flow cytometry at day 7, as both CD206 and arginase-1 expression peaks during the injury phase of the bleomycin^25,26^. In addition to anti-CD206 and anti-arg1, we included anti-CD45, anti-CD11b and anti-F4/80 to further characterize the AAM population. A total number of viable cells and the frequency of the population of interest was used to extrapolate absolute numbers. Consistent with the immunohistochemical assessment of arginase-1 positive cells described above, an increase of CD206+Arg1+ macrophages were found in lungs exposed to bleomycin plus AdOSM or AdIL-6 **(**Fig. 4A **)**. Gene expression and immunofluorescence assessment of macrophages isolated from the alveolar spaces indicated that mice exposed to both IL-6 and bleomycin had increased levels of *arginase-1* mRNA (Fig. 4B) and protein (Fig. 4C) as compared to mice receiving control vector only. As it was recently shown that IL-6 mediates alternative activation of macrophages through the induction of *Il-4rα* ^21^, we confirmed that *Il-4rα* gene expression was increased in alveolar macrophages (AMs) derived from AdIL-6 exposed lungs at Day 7 (Fig. 4D). Of note, mice receiving AdIL-6 alone did not show arginase-1 positive cells (data not shown).

### IL-6, but not OSM, directly acts on macrophages to induce M2 macrophage activation

**Figure 5:**
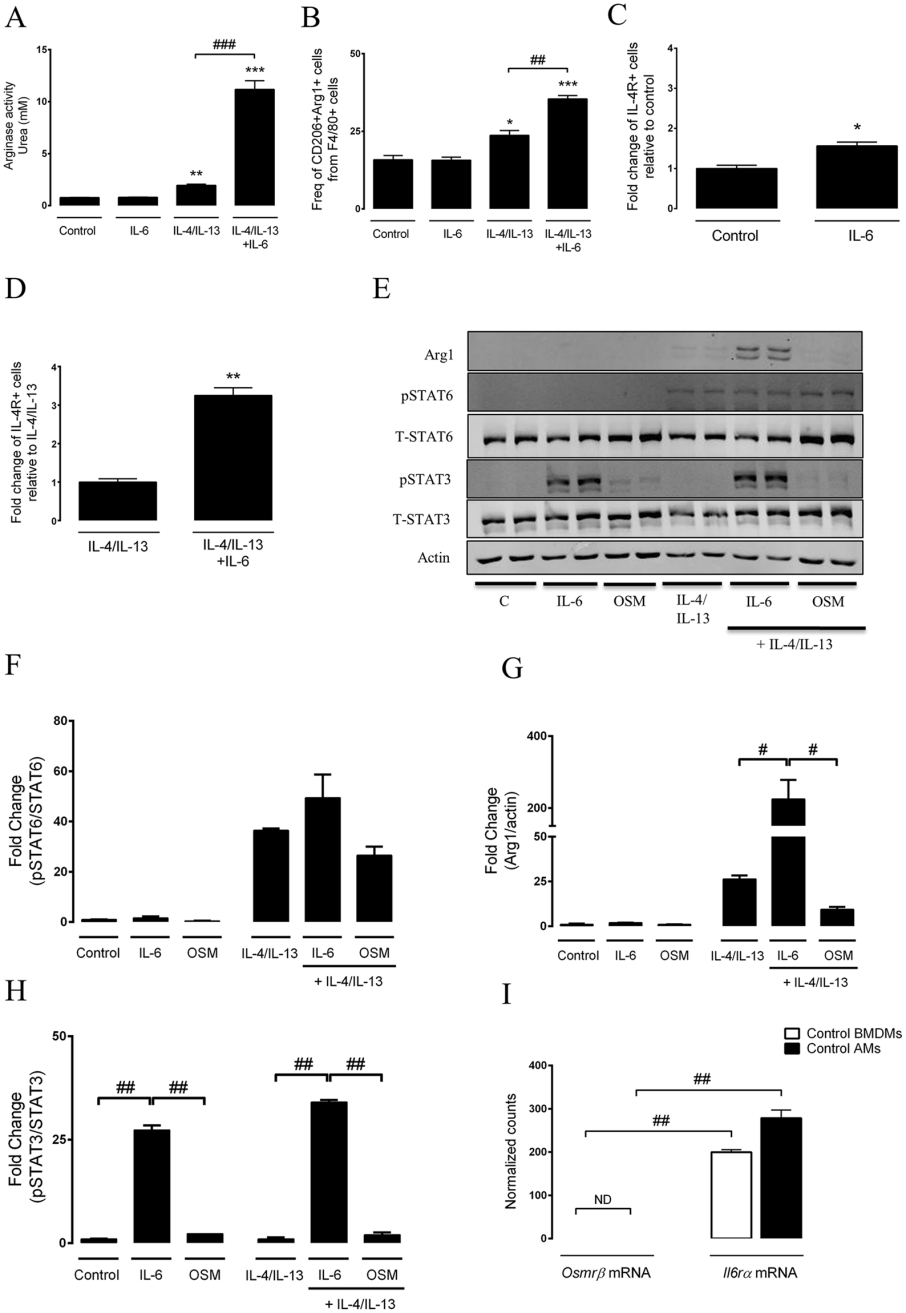
IL-6, but not OSM, directly acts on macrophages to potentiate their alternatively activated phenotype. Bone-marrow derived macrophages were cultured from naïve C57BL/6 for either 18 or 30 hours with recombinant OSM, IL-6, IL-4/IL-13, alone or in combination as indicated. AMs were isolated from naïve mice by adherence. BMDMs were either lysed and processed for (**A**) arginase activity assay, or analysed by flow cytometry to show (**B**) percentage of arg1+CD206+ macrophages, (**C**–**D**) fold change of percentage of IL-4Rα+ cells from the arg1+CD206+ population relative to controls. At least 100,000 events were captured per condition, and repeated twice. (**E**) Western blot analysis of cell lysates probed for arginase-1, pSTAT6, pSTAT3, and Actin. (**F**) Densitometry of pSTAT6 (corrected to STAT6), (**G**) arginase-1 (corrected to actin) and (**H**) pSTAT3 (corrected to STAT3) represented as a fold-change relative to control. (**I**) Normalized mRNA counts of *Osmrβ* and *Il6rα* from control (unstimulated) BMDMs and AMs. Flow cytometry and western blot results are from experiments completed in duplicates. The appropriate Western blot area depicting the antibody band was cropped and enclosed by black boxes, as indicated above. All samples were derived at the same time and processed in parallel. For Nanostring gene expression, lower than 5 counts was considered not detected “ND”. Bar graphs represent mean ± SEM from 2-3 replicates per group (showing one of two representative experiments), *P < 0.05; **P < 0.01; ***P < 0.001; ^#^P < 0.05; ^##^P < 0.01; ^###^P < 0.001; *represent a difference between any sample relative to the control (control vs. IL-4/IL-13, IL-6 vs. IL-4/IL-13 + IL-6); ^#^represent a difference between the indicated groups. Significance was established using GraphPad, Prism 7.0 with One-way ANOVA and non-parametric independent Student’s t-test.

To further investigate the molecular mechanisms involved in the alternative programming of macrophages, we resorted to using bone marrow-derived macrophages for the following reasons. a) primary alveolar macrophages do not have the capacity to polarize *in vitro* (data not shown); b) alternatively activated pro-fibrotic macrophages were recently shown to originate from the bone marrow^27^. Figure 5A shows a 5.7-fold increase in arginase activity when IL-6 is added to the conventional alternative programming cocktail, IL-4 and IL-13, which resulted in a 2.5-fold increase compared to non-stimulated cells. The administration of IL-6 alone did not have any effect on polarization in this assay. Importantly, the increased arginase activity was associated with increased levels of CD206+Arg1+ cells, as assessed by flow cytometry **(**Fig. 5B **)**. These data were consistent with the findings in lungs of mice exposed to bleomycin plus IL-6 shown above. We then assessed their ability to express IL-4Rα. IL-6 alone, or in combination with IL-4/IL-13, increased IL-4Rα expression on CD206+Arg1+ macrophages (Fig. 5C,D). To further examine the mechanism associated with IL-6 and OSM signalling, we stimulated BMDMs with IL-4/IL-13, alone or in combination with OSM or IL-6 for 18 hours and assessed STAT3 activation. Cell lysates were probed for arginase-1, phosphorylated STAT6 and phosphorylated STAT3 by Western Blot (Fig. 5E). Arginase-1 and phospho-STAT6 were induced in BMDMs following stimulation with IL-4/IL-13 (Fig. 5E–G). These results are consistent with previous studies demonstrating that Th2 cytokines, IL-4/IL-13, can activate the STAT6 pathway and induce an M2 macrophage phenotype^28–30^. Interestingly, IL-6 in combination with IL-4 and IL-13, (but not IL-6 alone) lead to robust increase in arginase-1 protein signal (Fig. 5E and G). These findings demonstrate the capacity of IL-6 to enhance the M2 macrophage polarization process. Activation of the STAT3 pathway was also observed by BMDMs stimulated with IL-6, but not OSM, suggesting that IL-6 may directly activate these cells (Fig. 5E and H **)**. For full-length pictures of western blots, please see supplementary Figure 10–12. The inability of OSM to augment phospho-STAT3 production in the BMDMs was consistent with mRNA data demonstrating differential receptor expression **(**Fig. 5I **)**. The mRNA for the IL-6 receptor, *Il-6rα*, was highly expressed in control unstimulated BMDMs and AMs, while mRNA counts for the OSM receptor (*Osmr*) were below the detection limit. Of note, when we looked at *Il-6rα* and *Osmr* expression in IL-4/IL-13, IL-4/IL-13/IL-6 stimulated BMDMs as well as bleomycin-exposed AMs, similar results were observed (see supplementary material, Fig. 3A,B). To examine Osmrβ expression profile on various macrophage ontology groups within the lung, we used publically available dataset obtained from Misharin *et al*.^31^. The log2-transformed counts demonstrate that Osmrβ was barely expressed on the different macrophage populations within the lung (see supplementary material, Fig. 3C). These findings suggest that OSM is likely not acting directly on BMDMs or AMs to induce changes in phenotype.

### IL-6 potentiates the pro-fibrotic phenotype of M2 macrophages

**Figure 6:**
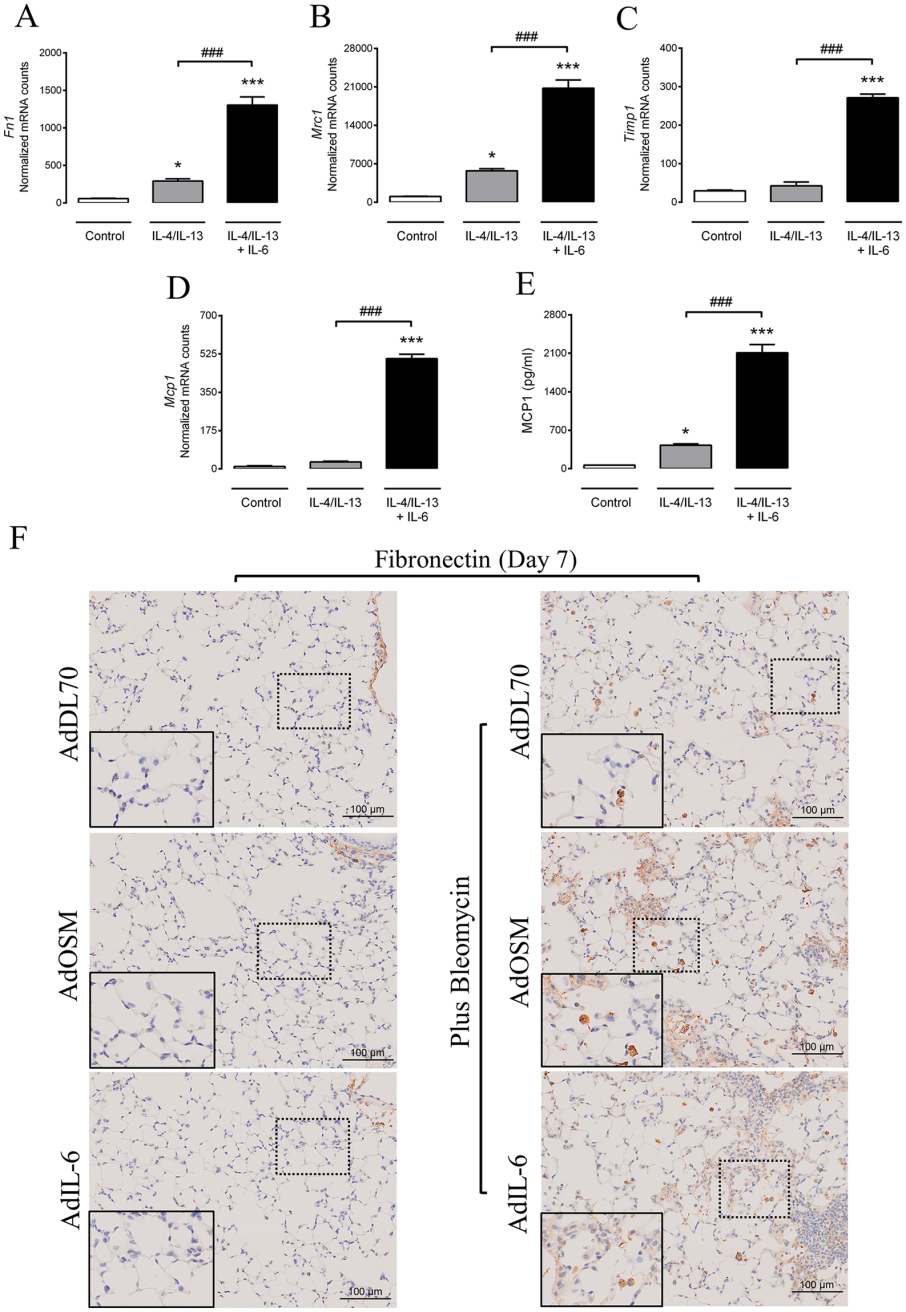
IL-6 increases the expression of pro-fibrotic factors in IL-4/IL-13 stimulated BMDMs. BMDMs were cultured from naïve C57BL/6 for 30 hours with recombinant IL-6 plus IL-4/IL-13. The harvested RNA and cellular supernatant were later examined for gene expression and cytokine/chemokine mediators, respectively. Anti-fibronectin antibody was used to stained lung tissues (Day 7). Significantly regulated pro-fibrotic related genes include (**A**) *Fn1* (**B**) *Mrc1* (**C**) *Timp-1 and* (**D**) *Mcp-1*. Analysis of mediators showed (**E**) MCP-1 protein level to be differentially regulated. (**F**) Representative images showing fibronectin positive staining. Bar graphs represent mean ± SEM from 3–5 samples per group, *P < 0.05; **P < 0.01; ***P < 0.001; ^#^P < 0.05; ^##^P < 0.01; ^###^P < 0.001; *represent a difference between any sample relative to the control (control vs. IL-4/IL-13 or IL-4/IL-13/IL-6); ^#^represent a difference between the indicated groups. Significance was established using GraphPad, Prism 7.0 with One-way ANOVA using Newman-Keuls Multiple Comparison test.

To examine the potential pro-fibrotic effect of IL-6 addition to the conventional M2 polarization cocktail (IL-4/IL-13), we performed gene expression analysis of selected pro-fibrotic related genes. Subsequently, we assessed 32 different mediators in the cell culture supernatant using a mouse cytokine/chemokine array. Nanostring gene expression analysis indicated that the addition of IL-6 to IL-4/IL-13 led to a robust increase in the pro-fibrotic related genes fibronectin 1 (*Fn1*), mannose receptor 1 (*Mrc1*), tissue inhibitor of metalloproteinase 1 (*Timp1*) and monocyte chemoattractant protein 1 (*Mcp-1*) **(**Fig. 6A–D **)**. Of note, both *Col1a1* and *Col3a1* were undetected in the IL-4/IL-13 condition, with or without IL-6 (data not shown). The multiplex array data showed that the assessed pro-inflammatory related cytokines were undetected in IL-4/IL-13/IL-6 exposed BMDM’s while secreted MCP-1 was strongly increased, confirming the gene expression data **(**Fig. 6E **)**. While fibronectin staining was absent in the parenchyma in lungs of control mice, robust staining was observed in areas of parenchymal injury and repair as well on cells that appeared to be “macrophage-like” cells 7 days after bleomycin and AdIL-6 or AdOSM adenoviral exposure (Fig. 6F; see also supplementary material, Fig. 4). At day 21 in the model, the majority of fibronectin staining was observed in the ECM in fibrotic areas (see supplementary material, Fig. 5). Of note, in all conditions and as expected, fibronectin staining was also seen around the airways and blood vessels, but an additive effect of staining was observed in mice exposed to bleomycin plus AdOSM or AdIL-6 compared to mice exposed to bleomycin and control vector. Overall, these data suggest unique phenotypic differences between IL-4/IL-13 (M2) - and IL-4/IL-13/IL-6- stimulated macrophages and different intensity of fibronectin staining *in vivo*.

## Discussion

**Figure 7:**
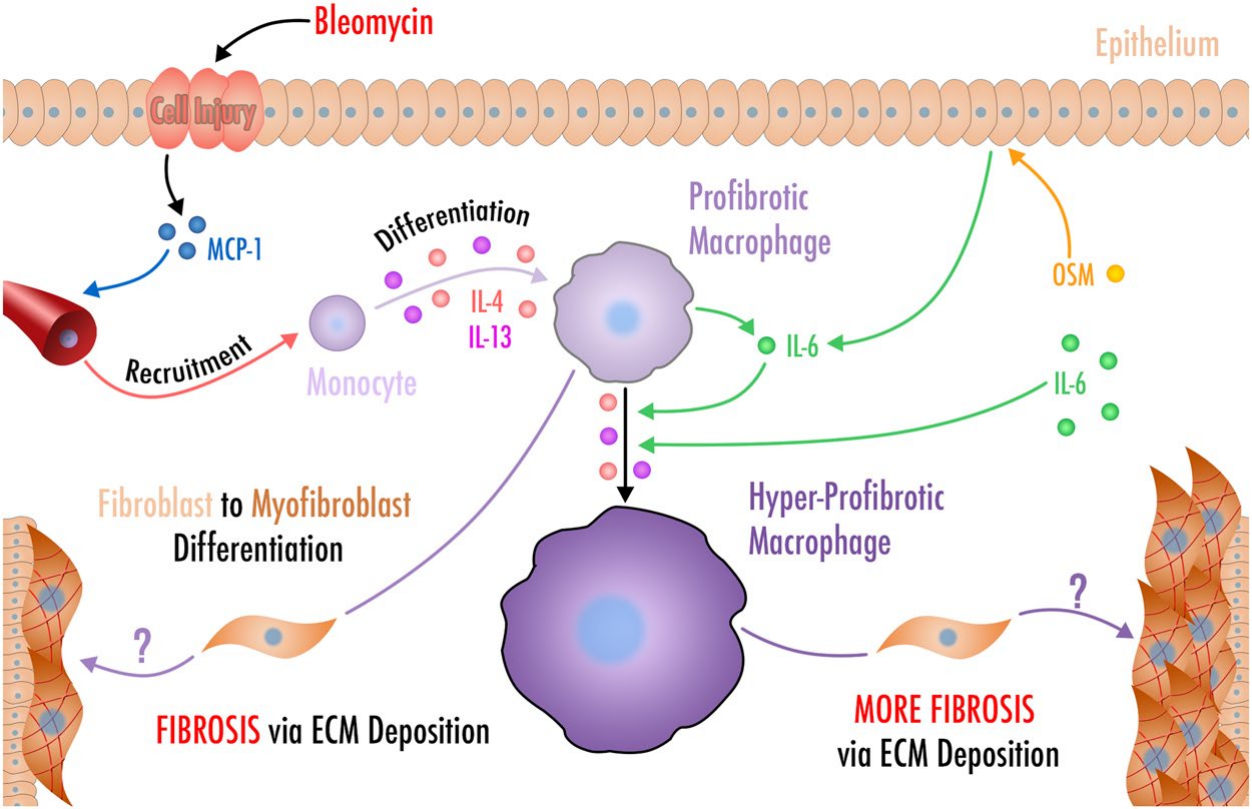
An illustration suggesting OSM or IL-6 presence in the lung acts as stimuli to further potentiate fibrotic disease development. Bleomycin exposure initiate epithelial injury, leading to the recruitment of immune cells and pro-fibrotic alternative macrophage polarization. Our data suggest that both gp130 cytokines, OSM or IL-6, have the capacity to enhance the fibrotic response to bleomycin, associated with an increased number of alternatively activated macrophages. We show further that IL-6 have the capacity to act directly on pulmonary macrophages as opposed to OSM, as macrophages do not express the OSM receptor.

Overall, we have shown that the gp130 cytokines, OSM and IL-6, potentiate bleomycin-induced lung injury and fibrosis. Overexpression of OSM or IL-6, in combination with bleomycin, resulted in an increase in lung elastance, collagen content and fibrotic score. Additionally, increased expression of these cytokines led to an increase in pulmonary M2-like macrophage accumulation *in vivo*, as demonstrated by induction of arginase-1 and CD206 expression. Our *in vitro* studies indicate that the enhancement of the M2-like phenotype is likely due to a direct activation of membrane-bound IL-6Rα by IL-6 and is associated with the activation of the STAT3 pathway as shown earlier^21^, in addition to the required STAT6 pathway activated by IL-4 and IL-13. The ability of IL-6 to directly augment the production of pro-fibrotic factors in the macrophage population suggests a possible *in vivo* pro-fibrotic function of the CD206+Arg1+ macrophage population. Together, these results suggest a novel role of gp130 cytokines signalling in modulating macrophage function and fibrogenesis that may be relevant for patients diagnosed with fibrotic lung disease (please see Fig. 7 for a cartoon summarizing the main finings of this study).

Although enhanced levels of OSM and IL-6 have previously been implicated in models of fibrotic disease^32–35^, our findings indicate that the overexpression of these cytokines alone (at the doses used) did not contribute to a fibrogenic response. Instead, we propose that bleomycin-induced injury primes the lung to OSM- and IL-6-driven pro-fibrotic functions. In the lungs exposed to either AdIL-6 or AdOSM and bleomycin, the high level of IL-6 that coincided with a marked increase in TGFβ1, could also suggest a crosstalk between the two cytokines as proposed by previous studies^36–38^. For example, it is possible that the high level of BALF IL-6 is augmenting the trafficking of TGFβ1 receptors to the cell surface and is, therefore, enhancing TGFβ1 Smad signalling on fibroblasts^36^. Alternatively, it would also be possible that TGFβ1 acts directly on fibroblasts to mediate an endogenous IL-6 release^37,38^, in turn contributing to enhanced macrophage programming. Although these explanations could potentially explain the increased TGFβ1 and IL-6 pro-fibrotic effects observed in our system, additional investigations would be required to imply a causative role in the pathogenesis of fibrosis. It was evident that a comparable level of induction of M2-like (Arg1+) population was observed in lungs exposed to both cytokines plus bleomycin even though AdOSM-induced levels of IL-6 were found to be almost 10-fold lower than those in mice exposed to AdIL-6. One plausible explanation for this phenotype could be attributed to the ability of OSM to increase IL-4 expression, as we have shown earlier^39^. In the present work, a lower dose of adenovirus vectors was used to induce a significant but limited elevation of cytokines. This change in dose reflects the absence of pathology observed in the AdOSM-treated mice, compared to our previous work^40^.

Although M2-like macrophages have been implicated in the fibrotic process, there is still ambiguity on the uniqueness of the markers that can precisely depict the origin and function of pro-fibrotic macrophages. Here we show that increased numbers of CD206+Arg1+ are associated with increased severity of pathology in the lung. However, it is not clear yet if these macrophages stemmed from the circulatory pool and if they are required for fibrogenesis to occur. Although CD206 is considered a marker of M2-like macrophages and that recently, *Satoh et al*. elegantly demonstrated that bone marrow-derived atypical monocytes expressed CD206 were required for fibrogenesis^27^, some reports suggest that naïve AMs may also express CD206^41^. Therefore, we cannot exclude the possibility that some CD206 resident macrophages underwent alternative activation in the bleomycin-exposed lungs, as opposed to increased infiltration of circulatory monocytes that were subsequently activated within the local tissue environment. The role of arginase-1 positive macrophages in fibrogenesis is debated. We showed recently that UPR-mediated apoptosis in the M2-like macrophage population was associated with decreased arginase-1 expression in the lung and complete protection from bleomycin-induced fibrosis^15^. However, Pesce *et al*. elegantly demonstrated that conditional arginase-1 deletion in the myeloid linage resulted in exacerbated *Schistosoma mansoni*-induced fibrosis, questioning the functional role of arginase-1 in the fibrotic process^42^. To have a better understanding of the exact role of CD206+Arg1+ macrophages in lung fibrosis, selective deletion strategies need to be developed and evaluated.

Previous studies have implicated both classical and trans-signalling of IL-6 in mediating the pathogenesis of fibrosis; Thanh-Thuy *et al*., demonstrated that blockade of IL-6 trans-signalling using the gp130 Fc antagonist attenuates bleomycin-induced lung fibrosis^20,43^. However, only classical signalling, mediated by direct IL-6 activity on membrane-bound IL-6Rα, was responsible for the enhanced alternative activation of macrophages in the presence of IL-4^21^. Our data demonstrate that unlike the OSMRβ, which was undetected on pulmonary macrophages and BMDMs, the IL-6Rα was abundantly expressed. Furthermore, based on the robust ability of IL-4/IL-13/IL-6-exposed macrophages to augment the induction of AAM markers such as arginase-1, arginase activity, Fn1, Mrc1, Timp1 and Mcp1, we refer here to these as “hyperpolarized” AAM. As such, the findings that IL-6 leads to a hyperpolarized macrophage, as opposed to OSM, suggest that OSM likely acts indirectly on macrophages in mice exposed to bleomycin. The high level of fibronectin expression *in vitro* (IL-4/IL-13/IL-6 mediated) and the enriched ECM-linked fibronectin seen in the fibrotic lungs after suggesting that these macrophages are likely contributors of the deposited fibronectin, as previously described^44,45^. The increased expression of IL-4Rα on the macrophage-enriched BALF cells and BMDMs in response to IL-6 stimulation was likely rendering monocytes/macrophages more sensitive to alternative programming by IL-4/IL-13. When overexpressed *in vivo*, both OSM and IL-6 led to increased STAT3 activation, as examined on whole lung tissues. Our *in vitro* data showed that only IL-6 induced STAT3 activation in BMDMs, as opposed to OSM. Thus, it is tempting to speculate that the difference observed in the STAT3 signalling activation *in vivo* and *in vitro* could be attributable to the selective localization of IL-6 and OSM receptors within different cell-types. It was previously shown that OSMRβ is primarily located on stromal or epithelial cells^17,46,47^, whereas IL-6Rα (via IL-6) is present on leukocytes^48^.

Overall, the findings from these experiments suggest that IL-6Rα, IL-4Rα and OSMRβ are potential cellular targets to reduce macrophage polarization and fibrotic disease progression. Although IL-4 is known to directly stimulate fibroblast proliferation and activation *in vitro* ^49,50^, its role *in vivo* is less clear. A previous study has shown that reduction of IL-4- and IL-13-responsive macrophages and mononuclear cells using IL-13-*Pseudomonas* exotoxin *A* fusion protein attenuates bleomycin-induced lung fibrosis in mouse models^51^. Additionally, therapeutic blockade of IL-4 in a mice model of scleroderma leads to reduced dermal collagen deposition and fibrosis^52^. However, further experimentation is needed to validate the use of anti-IL-6Rα, anti-IL-4Rα and anti-OSMRβ in disease models driven primarily by M2-like macrophages. The effect of the addition of IL-6 may also be relevant to better understand the molecular mechanisms of acute exacerbations in IPF (AE-IPF)^53^ as IL-6 has been indicated as a putative prognostic marker for AE-IPF^54,55^. AE-IPF associated mortality occurs in over 70% of patients with biopsy-confirmed IPF and over 90% in patients requiring ventilator assistance^53,56^. Various pro-inflammatory cytokines are elevated in the BALF in response to bacterial and viral infections, which are hypothesized to contribute to acute exacerbation^57^ and could constitute a valid therapeutic target to prevent progression of disease. Schupp *et al*. recently demonstrated that mechanisms regulating M2-like macrophage polarization could act as triggers of pathological wound healing and repair processes in patients with acute exacerbation of IPF^58^. While IL-6 may also elicit its action on fibroblasts to modulate fibrotic lung disease^59^, its direct involvement in alternative macrophage activation in the context of bleomycin-induced lung injury yields a novel and a potentially clinically relevant pathophysiological property of IL-6. This is consistent with the beneficial use of IL-6 neutralizing antibodies that were previously demonstrated to influence fibrotic outcomes during bleomycin-induced lung injury. Here, Kobayashi *et al*. showed that IL-6 blockade during the injury phase prevented the apoptosis of type II pneumocytes and markedly accelerated fibrosis, while IL-6 blockade at the early fibrotic phase ameliorated lung fibrosis. Of note, transient overexpression of IL-6 or OSM using the adenoviral delivery system, leads to a gradual increase in pulmonary transgene expression until approximately day 7-10, potentially explaining the differences observed by Kobayashi *et al*. Our data suggests that IL-6 alone does not impact the M2 polarization of macrophages, both *in vivo* and *in vitro*. As bleomycin exposure creates a TH2-like pro-fibrotic milieu, we believe that the addition of IL-6 synergizes with this milieu to enhance alternative programming and exacerbate fibrotic disease (please see Fig. 7). Combined, our data suggest that increased levels of OSM and IL-6 may be involved in promoting the accumulation of alternatively activated macrophages and subsequently fibrogenesis. As the bleomycin model is a reversible model, how the IL-6/OSM axis contributes to fibrotic disease resolution and whether it impacts the phenotypic characterization of macrophage subtypes would be an important avenue to consider in future studies. Therapeutic strategies targeting these cytokines or their receptors may be beneficial to limit the accumulation of pro-fibrotic macrophages and to prevent progression of fibrotic lung disease.

## Methods

### Animals and administration of bleomycin and adenoviral vectors

8–12 week wild type female C57BL/6 mice were purchased from Charles River Laboratories and housed in the Central Animal Facility. The animals were kept on a 12 h light/12 h dark cycle at a temperature of 20–25 °C, humidity of ~50% and fed *ad libitum*. All work was conducted under the guidelines of the Canadian Council on Animal Care and approved by the Animal Research Ethics Board of McMaster University under Protocol #160414. Transient overexpression of OSM or IL-6 was achieved using adenovirus expression vectors, AdOSM and AdIL-6. Vector construction and validation were performed in-house based on previously published work^60–62^. Briefly, mice were anaesthetized under gaseous isoflurane while AdOSM (2 × 10^7^ pfu/mouse) and AdIL-6 (1 × 10^7^ pfu/mouse) was intratracheally administered in a volume of 50 μl sterile saline. Control mice received an empty control vector, AdDL70 (2 × 10^7^ pfu/mouse). Experimental pulmonary fibrosis was induced using intratracheal intubation of bleomycin (Hospira Healthcare Corp., NDC 61703-332-18) at 0.03 U/mouse in a volume of 50 µl sterile saline. Bleomycin was either given alone or with AdOSM, AdIL-6 or AdDL70. The mice were then sacrificed after 7 days (injury phase) and 21 days (fibrotic phase).

### Airway Physiology measurements

Quasi-static lung elastance was measured using a rodent mechanical ventilator (flexiVent^®^, SCIREQ, Montreal, PQ, Canada) as described earlier^15^.

### Collection of mouse specimens

Mice were anesthetized with gaseous isoflurane and exsanguinated by surgically severing the descending aorta. The trachea was cannulated, the lung excised and washed with phosphate-buffered saline (600 µl per mouse) for broncheoalveolar lavage fluid (BALF) collection and analysis. The four lobes of the right lung were tied up with a surgical thread, excised and either placed in cold DMEMF12 media or frozen in liquid nitrogen. These lobes were enzymatically digested to generate a single cell suspension and subsequently stained for flow cytometry. The frozen lobes were subjected to protein and RNA isolation assays. The left lung was removed and inflated with 10% neutral buffered formalin solution under a pressure of 30 cmH_2_O. The lungs were fixed for 48–72 hours before histological analysis.

### Homogenization of lung tissues

The snap-frozen right lobes of the excised lungs were placed into a stainless-steel mortar and crushed into a fine powder with a stainless-steel pestle while submerged in liquid nitrogen. Approximately one-third of each finely ground lung tissue was placed in 1 ml of TRIzol reagent (Invitrogen, cat# 15596-026), with two-thirds being placed into 1 ml (RIPA) lysis buffer supplemented with protease inhibitors (1 × PBS, 1% IGEPAL CA-130, 0.5% Na-deoxycholate, 0.1% SDS and 1 mM Na-orthovanadate, 5 µg/ml aprotinin, 1 mM phenylmethylsulphonylfluoride and 1 mM dithiothreitol). The powdered lungs in the lysis buffer and TRIzol solutions were then mechanically sheared using a homogenizer (Ultra-Turrax^®^ T25) and later processed for protein and RNA work.

### Western Blotting

Protein concentration of lung homogenates or cell lysates was measured by Bradford Assay (Bio-Rad). Samples were prepared with 15 or 20ug of total protein, ddH2O and 5x loading dye, for a final volume of 20uL. 4-20% Tris-Glycine Sodium deoxysulfate-polyacrylamide gels (SDS-PAGE) were prepared fresh and used immediately or stored at 4 °C overnight. Samples were loaded onto gels and run at 95 V for 12 minutes, then at 120 V for 1 hour by electrophoresis. Protein was then transferred onto nitrocellulose membranes at 400 mA for 1 hour. Blocking buffer (1:1 TBS: Odyssey blocking Buffer) was added to membranes for 1 hour at room temperature. Following blocking, primary antibodies including mouse anti-arginase-1 (BD Biosciences, cat# 610708), rabbit anti-pSTAT3 (Cell Signaling, Cat# 9145), rabbit anti-total STAT3 (Cell Signaling, Cat# 4904), rabbit anti-pSTAT6 (Cell Signaling, Cat# 9361), rabbit anti-total STAT6 (Santa Cruz Biotechnology, Cat# sc-981) and goat anti-actin (Santa Cruz Biotechnology, Cat# sc-1616) were added to blots overnight at 4 °C. Blots were then washed using TBS + 0.15% Tween-20, and secondary antibodies (donkey anti-goat (Mandel Scientific, Cat# LIC92668074), Donkey anti-rabbit (Mandel Scientific, Cat# LIC92632213), or Donkey anti-mouse (Mandel Scientific, Cat# LIC92632212)) were added for 45 minutes, away from light. Blots were then imaged using Odyssey LI-COR® Imaging technologies. Quantification of protein signals were measured by densitometry using Image Studio Lite Ver 5.2.

### Immunohistochemistry

Immunohistochemistry was performed at McMaster’s Histology Core Facility as previously described^15^. Briefly, formalin-fixed tissues were cut at 5 µm sections and stained with Masson’s trichrome for ECM, anti-αSMA (Dako, cat# M0851) for the identification of αSMA-positive myofibroblasts and anti-fibronectin (Abcam, cat# ab2413) for the assessment of pro-fibrotic factors. Lung tissue sections were also stained with anti-arginase-1 (BD Biosciences, cat# 610708) for the identification of alternatively activated macrophages. We used the Dako ARK^TM^ (Animal Research Kit) Peroxidase (cat# K3954) to execute immunohistochemical stains involving mouse primary antibodies on mouse lung tissues. As for tissues that contain endogenous biotin (such as liver and kidney and other tissues), a step involving avidin/biotin block (Vector Laboratories, cat# SP2001) was conducted prior to immunohistochemical staining of the lung tissues. Diluent and IgG negative controls as well as non-treated tissue controls were included to ensure precision of the staining protocol and optimal antibody concentrations.

### Immunofluorescence

Immunostaining of arginase-1 was performed on BALF cellular infiltrates mounted on cytospin slides. Briefly, BALF cells were first fixed in 4% paraformaldehyde solution, followed by permeabilization with 0.1% Triton X-100 PBS solution and subsequent blocking with 5% BSA solution. Cells were then incubated with anti-arginase-1 antibody (BD Biosciences, cat# 610708) for 1 hour before the application of a secondary antibody (Donkey anti-mouse Alexa 488 (Abcam, Cat# Ab150105) for 30 minutes. Slides were mounted in Prolong-gold/ DAPI before imaging.

### Acquisition and analysis

Microscope slides were digitalized using an Olympus VS120-L100-W slide scanner microscope, and subsequent images were acquired to illustrate representative areas. Semi-quantitative assessment of the severity of fibrosis using the Ashcroft grading procedure was performed based on Masson’s trichrome staining, as described previously^15^. For αSMA, the main bronchus and larger airways were excluded by ImageJ. For both arginase-1 and αSMA, once thresholding was applied to specifically display the stained regions, the analyze particle function was then used to determine the total area of the stained regions. Image analysis was performed on image J to demonstrate the percentage of arginase-1 and αSMA staining normalized to the whole tissue area.

### Lung single cell isolation and purification

Following sacrifice, the lungs were isolated, minced into small pieces and digested with 150 U/ml collagenase type I (Gibco, Cat# 17100-017) in warm DMEMF12 media for 1 h at 37 °C with agitation. The digested lung pieces were then pushed through a 45 µm filter (BD Falcon) and the single cell suspension was treated with ACK lysis buffer for 1-2 min at 4 °C to lyse all erythrocytes. Lung cells were subsequently suspended in media and total number of viable cells and percent viability was determined using the Countess automated cell counter (Invitrogen).

### Flow cytometry

Approximately 2 × 10^6^ viable lung cells or bone marrow derived macrophages were suspended in cell staining buffer (0.3% BSA in PBS). Non-antigen-specific binding of immunoglobulins to Fcγ II/III receptor on lung cells was blocked using purified rat anti-mouse CD16/32 antibody (Mouse FC Block) (BD Pharmingen, Cat# 553142). Next, lung cells were subjected to surface antibody staining solution, containing a mix of anti-mouse CD45 (APC-Cy7, Biolegend, Cat#103116), anti-mouse CD206 (MMR) (BV650, Biolegend, Cat# 141723), anti-mouse CD11b (PE, eBiosciences, Cat# 12-0112-81) and anti-mouse F4/80 (BV605, Biolegend, Cat# 123133). BMDMs were stained with anti-mouse CD206, anti-mouse F4/80 and anti-mouse CD124 (IL-4Rα) (PE, BD Pharmingen, Cat# 552509). All cells were then fixed and permeabilized using a BD Cytofix/Cytoperm kit (BD Biosciences, Cat# 554722) prior to performing intracellular staining with anti-mouse/human arginase-1 (APC, R&D Systems, Cat# IC5868A) suspended in 1x BD Perm/Wash buffer solution (BD Biosciences, Cat# 554723). Cells were finally suspended in cell staining buffer for flow cytometry and data was collected using a BD LSRFortessa^TM^ and FACSDiva software from BD Biosciences. Data were analyzed using FlowJo vX Software from Treestar.

### Murine alveolar macrophages isolation

Following exposure to the above conditions, lungs were harvested and BALF was collected as described above. Collected cells were then cultured in Dulbecco’s Modified Eagle Medium (F12) containing 10% Fetal Bovine Serum. After 40 minutes in culture, adherent cells were selected by washing the non-adherent cell population. Macrophage-enriched cells were then lysed and RNA was isolated for nanoString gene expression analysis.

### Isolation and alternative activation of bone marrow-derived macrophages

Bone marrow-derived macrophages (BMDMs) were isolated and cultured as described previously^15^. Briefly, bone marrow cells from wild type C57BL/6 mice were isolated from the tibias and femurs and treated with 20 ng/ml of recombinant mouse macrophage colony stimulating factor (Murine M-CSF, PeproTech Canada) for 7 days. After 7 days, bone marrow derived macrophages were treated for either 18 or 30 hours with recombinant IL-4 (20 ng/ml), IL-13 (50 ng/ml), alone, or in combination with OSM (50 ng/ml) or IL-6 (50 ng/ml) (PeproTech Canada). Alternative activation of macrophages was assessed in the cell lysate by measuring arginase-1 protein by western blotting and by arginase-1 and CD206 by flow cytometry. In some instances, BMDMs were lysed and RNA was isolated for NanoString gene expression analysis.

### Arginase assay

The arginase assay was adapted and optimized from a previously established protocol^23^. Following the seeding of BMDMs (80,000 cells/per well of a 96-well plate) and their subsequent stimulation with the above cytokine cocktails, supernatant was collected and cells were washed twice with ice-cold PBS. Next, cells were lysed in 25-100 μl of 0.1% Triton X-100 supplemented with protease inhibitors (200 mM sodium orthovanadate, 0.1 M PMSF, 1 M DTT and 5 μg/ml and bovine lung aprotinin). A 1:1 dilution with 25 mM of Tris-HCl (pH 7.5) was later performed. 25 μl of this mixture was transferred to a 96-well PCR plate, with the addition of 2.5 μl of 10 mM manganese chloride. The PCR plate was then placed in a thermal cycler programmed to heat at 56 degrees Celsius for 10 minutes. Afterwards, 25 μl of 0.5 M L-arginine was mixed with the pre-heated mixture and the entire plate was then re-incubated at 37 degrees Celsius for 30 minutes. An eight-point urea standard curve was then established (0 to 15 mM). 200 μl of acid solution (63.6% water, 9.1% concentrated sulphuric acid and 27.3% concentrated phosphoric acid), followed by 10 μl of 9% alpha-isonitrosopropiophenone, were added to both samples and the urea solutions. The contents of each well were mixed thoroughly and the plate was incubated at 95 degrees Celsius for 30 minutes, followed by 10 minutes of cooling at 20 degrees Celsius. 150-200 μl of each sample was removed and placed in a new, clear, flat bottom, 96-well plate for absorbance reading at 550 nm.

### RNA isolation and nanoString gene expression

RNA was isolated from adhered alveolar macrophages and BMDMs using NucleoSpin RNA plus (Macherey Nagel, Cat# 740984.250). Here, we used a nanoString custom designed panel focusing on selected genes including Il4ra, Arg1, Ccl2, Il6ra, Timp1, Osmr, Col1a1, Col1a3, Fn1, Mrc1. Analysis of raw mRNA counts was performed using nSolver v2.6. Background subtraction was performed using geometric mean of negative controls, followed by normalization to geometric mean of positive controls. Results were analyzed and normalized to housekeeping genes (Actb, Pgk1, Ppia and Ywhaz) and with the nanoString total counts method as described in the nCounter Expression Analysis Guide, producing similar results. Gene expression data is here shown as normalized to total counts.

### GEO database analysis

Publically available dataset containing *Osmrβ* expression profiles of monocyte/macrophage populations were obtained Misharin *et al*.^31^. Briefly, following bleomcyin administration, wild type C57BL6 (Casp8flox/flox) mice were sacrificed after 14 and 19 days and lung cells were flow-sorted to isolate monocytes, interstitial macrophages, and alveolar macrophages (distinguished by high or low expression of Siglec F). For naïve mice, Siglec F high Alveolar macrophages were isolated. Normalized counts were log2-transformed, and then the distribution of the Osmr Log2-normalized counts was compared to the distribution of Log2-normalized counts pooled across all genes and all samples. Plots were built by using R environment.

### ELISA and Multiplex analysis of mediators

BALF total TGFβ1 was measured using a commercially available ELISA, according to the manufacturer’s protocol (R&D Systems, Cat# DY1679). We also quantified 32 different mediators in the BALF and cell culture supernatant by using a Discovery Assay® (Mouse Cytokine and Chemokine Array 32-Plex, Eve Technologies Corp, Calgary, AB, Canada). The multiplex assay was performed at Eve Technologies using the Bio-Plex™ 200 system (Bio-Rad Laboratories, Inc., Hercules, CA, USA) and a Milliplex Mouse Cytokine/Chemokine kit (Millipore, St. Charles, MO, USA) according to Evetech protocol. The 32-plex consisted of eotaxin, G-CSF, GM-CSF, IFN-γ, IL-1α, IL-1β, IL-2, IL-13, IL-4, IL-5, IL-6, IL-7, IL-9, IL-10, IL-12 (p40), IL-12 (p70), IL-13, IL-15, IL-17, IP-10, KC, LIF, LIX, MCP-1, M-CSF, MIG, MIP-1α, MIP-1β, MIP-2, RANTES, TNFα, and VEGF. The assay sensitivities of these markers range from 0.1 pg/ml to 33.3 pg/ml. Individual analytes’ values and other assay details are available on Eve Technologies’ website and in the Milliplex protocol.

### Sircol Collagen Assay

Following lung homogenization, the supernatant from the homogenized lung tissues in RIPA buffer was used to assess soluble lung collagen content, according to the manufacturer’s instructions (Sircol^TM^ Soluble Collagen Assay, Biocolor, UK).

### Statistical analysis

Results are expressed as mean ± SEM. A one-way analysis of variance (One-way ANOVA) followed by Newman-Keuls multiple comparison test was used to determine significance when more than two groups were compared. A student’s t-test was used to determine significance between two conditions. All statistical tests were performed using GraphPad Prism 7.0c (GraphPad Software, Inc). A p < 0.05 was considered statistically significant.

### Data availability

The datasets generated during and/or analysed during the current study are available from the corresponding author on reasonable request.

## Electronic supplementary material

Online supplement

## References

[1] Sgalla G, Biffi A, Richeldi L (2016). Idiopathic pulmonary fibrosis: Diagnosis, epidemiology and natural history. Respirology (Carlton, Vic.).

[2] Gifford AH, Matsuoka M, Ghoda LY, Homer RJ, Enelow RI (2012). Chronic inflammation and lung fibrosis: pleotropic syndromes but limited distinct phenotypes. Mucosal Immunol.

[3] Bringardner BD, Baran CP, Eubank TD, Marsh CB (2008). The role of inflammation in the pathogenesis of idiopathic pulmonary fibrosis. Antioxid Redox Signal.

[4] Ask, K., Martin, G. E., Kolb, M. & Gauldie, J. Targeting genes for treatment in idiopathic pulmonary fibrosis: challenges and opportunities, promises and pitfalls. *Proceedings of the American Thoracic Society* **3**, 389–393, 10.1513/pats.200602-021TK (2006). 10.1513/pats.200602-021TK 16738206

[5] Moeller A, Ask K, Warburton D, Gauldie J, Kolb M (2008). The bleomycin animal model: a useful tool to investigate treatment options for idiopathic pulmonary fibrosis?. The international journal of biochemistry & cell biology.

[6] Park S, Lee EJ (2013). Recent advances in idiopathic pulmonary fibrosis. Tuberculosis and respiratory diseases.

[7] Travis WD (2013). An official American Thoracic Society/European Respiratory Society statement: Update of the international multidisciplinary classification of the idiopathic interstitial pneumonias. Am J Respir Crit Care Med.

[8] Fernandez IE, Eickelberg O (2012). The impact of TGF-beta on lung fibrosis: from targeting to biomarkers. Proc Am Thorac Soc.

[9] Selman M, Pardo A, Richeldi L, Cerri S (2011). Emerging drugs for idiopathic pulmonary fibrosis. Expert Opin Emerg Drugs.

[10] Coward WR, Saini G, Jenkins G (2010). The pathogenesis of idiopathic pulmonary fibrosis. Therapeutic advances in respiratory disease.

[11] Gibbons MA (2011). Ly6Chi monocytes direct alternatively activated profibrotic macrophage regulation of lung fibrosis. American journal of respiratory and critical care medicine.

[12] Wynn, T. A. *et al*. Quantitative assessment of macrophage functions in repair and fibrosis. Current protocols in immunology/edited by John E. Coligan… [*et al*.] Chapter 14, Unit14 22, 10.1002/0471142735.im1422s93 (2011). 10.1002/0471142735.im1422s93 PMC3109612 21462164

[13] Wynn TA, Chawla A, Pollard JW (2013). Macrophage biology in development, homeostasis and disease. Nature.

[14] Wynn TA, Barron L (2010). Macrophages: master regulators of inflammation and fibrosis. Seminars in liver disease.

[15] Ayaub EA (2016). GRP78 and CHOP modulate macrophage apoptosis and the development of bleomycin-induced pulmonary fibrosis. J Pathol.

[16] Pedersen BK (2011). Muscles and their myokines. The Journal of experimental biology.

[17] Richards CD (2013). The enigmatic cytokine oncostatin m and roles in disease. ISRN inflammation.

[18] Silver JS, Hunter C (2010). A. gp130 at the nexus of inflammation, autoimmunity, and cancer. Journal of leukocyte biology.

[19] Sims NA, Walsh NC (2010). GP130 cytokines and bone remodelling in health and disease. BMB reports.

[20] O’Donoghue RJ (2012). Genetic partitioning of interleukin-6 signalling in mice dissociates Stat3 from Smad3-mediated lung fibrosis. EMBO molecular medicine.

[21] Mauer J (2014). Signaling by IL-6 promotes alternative activation of macrophages to limit endotoxemia and obesity-associated resistance to insulin. Nat Immunol.

[22] Komori T (2015). Oncostatin M is a potential agent for the treatment of obesity and related metabolic disorders: a study in mice. Diabetologia.

[23] Lauber S (2015). Novel function of Oncostatin M as a potent tumour-promoting agent in lung. Int J Cancer.

[24] West NR (2017). Oncostatin M drives intestinal inflammation and predicts response to tumor necrosis factor-neutralizing therapy in patients with inflammatory bowel disease. Nat Med.

[25] Sester DP (2015). A novel flow cytometric method to assess inflammasome formation. J Immunol.

[26] Endo M (2003). Induction of arginase I and II in bleomycin-induced fibrosis of mouse lung. Am J Physiol Lung Cell Mol Physiol.

[27] Satoh T (2017). Identification of an atypical monocyte and committed progenitor involved in fibrosis. Nature.

[28] Stein M, Keshav S, Harris N, Gordon S (1992). Interleukin 4 potently enhances murine macrophage mannose receptor activity: a marker of alternative immunologic macrophage activation. The Journal of experimental medicine.

[29] Nair MG, Guild KJ, Artis D (2006). Novel Effector Molecules in Type 2 Inflammation: Lessons Drawn from Helminth Infection and Allergy. The Journal of Immunology.

[30] Mylonas KJ, Hoeve MA, MacDonald AS, Allen JE (2013). Alternative activation of macrophages by filarial nematodes is MyD88-independent. Immunobiology.

[31] Misharin AV (2017). Monocyte-derived alveolar macrophages drive lung fibrosis and persist in the lung over the life span. The Journal of experimental medicine.

[32] Mozaffarian A (2008). Mechanisms of oncostatin M-induced pulmonary inflammation and fibrosis. J Immunol.

[33] Nagahama KY (2013). Oncostatin M modulates fibroblast function via signal transducers and activators of transcription proteins-3. Am J Respir Cell Mol Biol.

[34] Scaffidi AK (2002). Oncostatin M stimulates proliferation, induces collagen production and inhibits apoptosis of human lung fibroblasts. British journal of pharmacology.

[35] Saito F (2008). Role of interleukin-6 in bleomycin-induced lung inflammatory changes in mice. American journal of respiratory cell and molecular biology.

[36] Zhang XL, Topley N, Ito T, Phillips A (2005). Interleukin-6 regulation of transforming growth factor (TGF)-beta receptor compartmentalization and turnover enhances TGF-beta1 signaling. The Journal of biological chemistry.

[37] Eickelberg O (1999). Transforming growth factor-beta1 induces interleukin-6 expression via activating protein-1 consisting of JunD homodimers in primary human lung fibroblasts. The Journal of biological chemistry.

[38] Elias JA, Lentz V, Cummings PJ (1991). Transforming growth factor-beta regulation of IL-6 production by unstimulated and IL-1-stimulated human fibroblasts. J Immunol.

[39] Fritz DK (2011). A mouse model of airway disease: oncostatin M-induced pulmonary eosinophilia, goblet cell hyperplasia, and airway hyperresponsiveness are STAT6 dependent, and interstitial pulmonary fibrosis is STAT6 independent. J Immunol.

[40] Wong S, Botelho FM, Rodrigues RM, Richards CD (2014). Oncostatin M overexpression induces matrix deposition, STAT3 activation, and SMAD1 Dysregulation in lungs of fibrosis-resistant BALB/c mice. Laboratory investigation; a journal of technical methods and pathology.

[41] Hussell T, Bell TJ (2014). Alveolar macrophages: plasticity in a tissue-specific context. Nature reviews. Immunology.

[42] Pesce JT (2009). Arginase-1-expressing macrophages suppress Th2 cytokine-driven inflammation and fibrosis. PLoS Pathog.

[43] Le T-TT (2014). Blockade of IL-6 Trans Signaling Attenuates Pulmonary Fibrosis. The Journal of Immunology Author Choice.

[44] Gratchev A (2001). Alternatively activated macrophages differentially express fibronectin and its splice variants and the extracellular matrix protein betaIG-H3. Scandinavian journal of immunology.

[45] Rennard SI, Hunninghake GW, Bitterman PB, Crystal RG (1981). Production of fibronectin by the human alveolar macrophage: mechanism for the recruitment of fibroblasts to sites of tissue injury in interstitial lung diseases. Proceedings of the National Academy of Sciences of the United States of America.

[46] Cichy J, Rose-John S, Pure E (1998). Regulation of the type II oncostatin M receptor expression in lung-derived epithelial cells. FEBS letters.

[47] Tamura S, Morikawa Y, Tanaka M, Miyajima A, Senba E (2002). Developmental expression pattern of oncostatin M receptor beta in mice. Mechanisms of development.

[48] Hunter CA, Jones SA (2015). IL-6 as a keystone cytokine in health and disease. Nature immunology.

[49] Saito A, Okazaki H, Sugawara I, Yamamoto K, Takizawa H (2003). Potential Action of IL-4 and IL-13 as Fibrogenic Factors on Lung Fibroblasts *in vitro*. International Archives of Allergy and Immunology.

[50] Postlethwaite AE, Holness MA, Katai H, Raghow R (1992). Human fibroblasts synthesize elevated levels of extracellular matrix proteins in response to interleukin 4. Journal of Clinical Investigation.

[51] Jakubzick C (2003). Therapeutic attenuation of pulmonary fibrosis via targeting of IL-4- and IL-13-responsive cells. J Immunol.

[52] Ong C, Wong C, Roberts CR, Teh HS, Jirik FR (1998). Anti-IL-4 treatment prevents dermal collagen deposition in the tight-skin mouse model of scleroderma. European journal of immunology.

[53] Song JW, Hong SB, Lim CM, Koh Y, Kim DS (2011). Acute exacerbation of idiopathic pulmonary fibrosis: incidence, risk factors and outcome. The European respiratory journal.

[54] Collard HR (2010). Plasma biomarker profiles in acute exacerbation of idiopathic pulmonary fibrosis. Am J Physiol Lung Cell Mol Physiol.

[55] Bhatti H, Girdhar A, Usman F, Cury J, Bajwa A (2013). Approach to acute exacerbation of idiopathic pulmonary fibrosis. Annals of thoracic medicine.

[56] Collard HR (2007). Acute exacerbations of idiopathic pulmonary fibrosis. Am J Respir Crit Care Med.

[57] Chen WH (2012). Potential Role for Alternatively Activated Macrophages in the Secondary Bacterial Infection During Recovery from Influenza. Immunology letters.

[58] Schupp JC (2015). Macrophage Activation in Acute Exacerbation of Idiopathic Pulmonary Fibrosis. PLOS ONE.

[59] Hendrayani SF, Al-Khalaf HH, Aboussekhra A (2014). The cytokine IL-6 reactivates breast stromal fibroblasts through transcription factor STAT3-dependent up-regulation of the RNA-binding protein AUF1. The Journal of biological chemistry.

[60] Braciak TA, Mittal SK, Graham FL, Richards CD, Gauldie J (1993). Construction of recombinant human type 5 adenoviruses expressing rodent IL-6 genes. An approach to investigate *in vivo* cytokine function. J Immunol.

[61] Kerr C (1999). Adenovirus vector expressing mouse oncostatin M induces acute-phase proteins and TIMP-1 expression *in vivo* in mice. Journal of interferon & cytokine research: the official journal of the International Society for Interferon and Cytokine Research.

[62] Richards, C. D., Braciak, T., Xing, Z., Graham, F. & Gauldie, J. Adenovirus vectors for cytokine gene expression. *Annals of the New York Academy of Sciences* **762**, 282–292; discussion 292–283 (1995). 10.1111/j.1749-6632.1995.tb32333.x 7668531

