# Supplementary material for: Overexpression of OSM and IL-6 impacts the polarization of pro-fibrotic macrophages and the development of bleomycin-induced lung fibrosis
Source: Sci Rep. 2017 Oct 16;7:13281. doi: 10.1038/s41598-017-13511-z (PMC5643520; doi:10.1038/s41598-017-13511-z)
Supplement: Supplementary file 1 — Online supplement [file 41598_2017_13511_MOESM1_ESM.pdf]

Supplementary Information

Overexpression of OSM and IL-6 impacts the polarization of pro-fibrotic macrophages and the development of bleomycin-induced lung fibrosis

Ehab A Ayaub[1,2], Anisha Dubey [2], Jewel Imani [1,2], Fernando Botelho[2], Martin RJ Kolb [1], Carl D Richards[2], Kjetil Ask *[1,2]

1. *Department of Medicine, Firestone Institute for Respiratory Health, McMaster University and The Research Institute of St. Joe's Hamilton, Hamilton, ON, Canada*
2. *Department of Pathology and Molecular Medicine, McMaster Immunology Research Centre, McMaster University, Hamilton, ON, Canada*

***Corresponding author:** Dr. Kjetil Ask, Department of Medicine, McMaster University and The Research Institute of St. Joe's Hamilton, Firestone Institute for Respiratory Health, Luke Wing, Rm L314-5, 50 Charlton Ave East, Hamilton, Ontario, Canada L8N 4A6, Ph. (905) 522 1155 ext. 33683; Fax (905) 521 6183; E-mail: askkj@mcmaster.ca

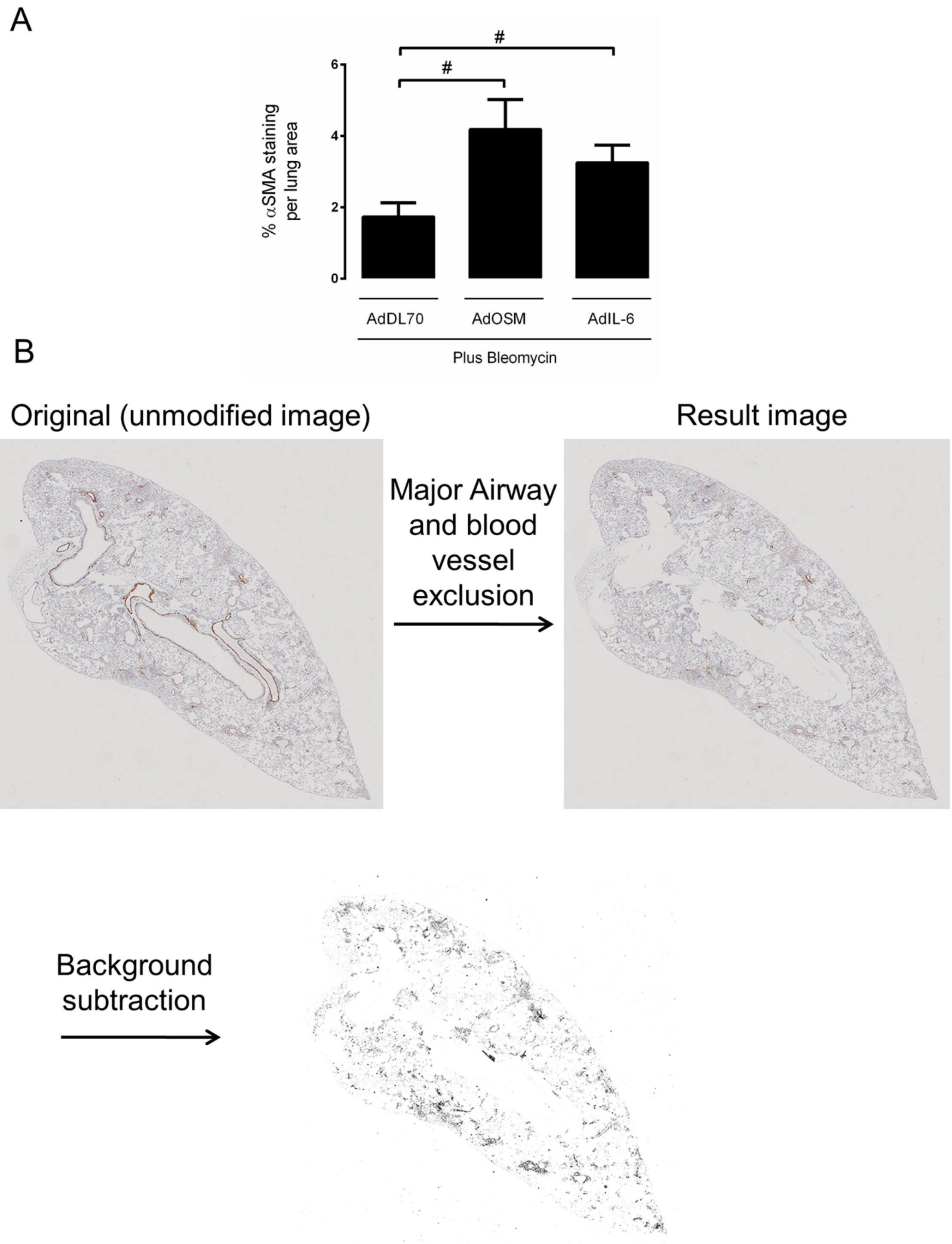

Supplementary Figure 1. Quantification of parenchymal αSMA staining in bleomycin-exposed lungs. Following the exclusion of major airways and blood vessels, (A) parenchymal αSMA was assessed and shown as a percentage of the lung region. (B) Example of airway/vessel exclusion and thresholding method.

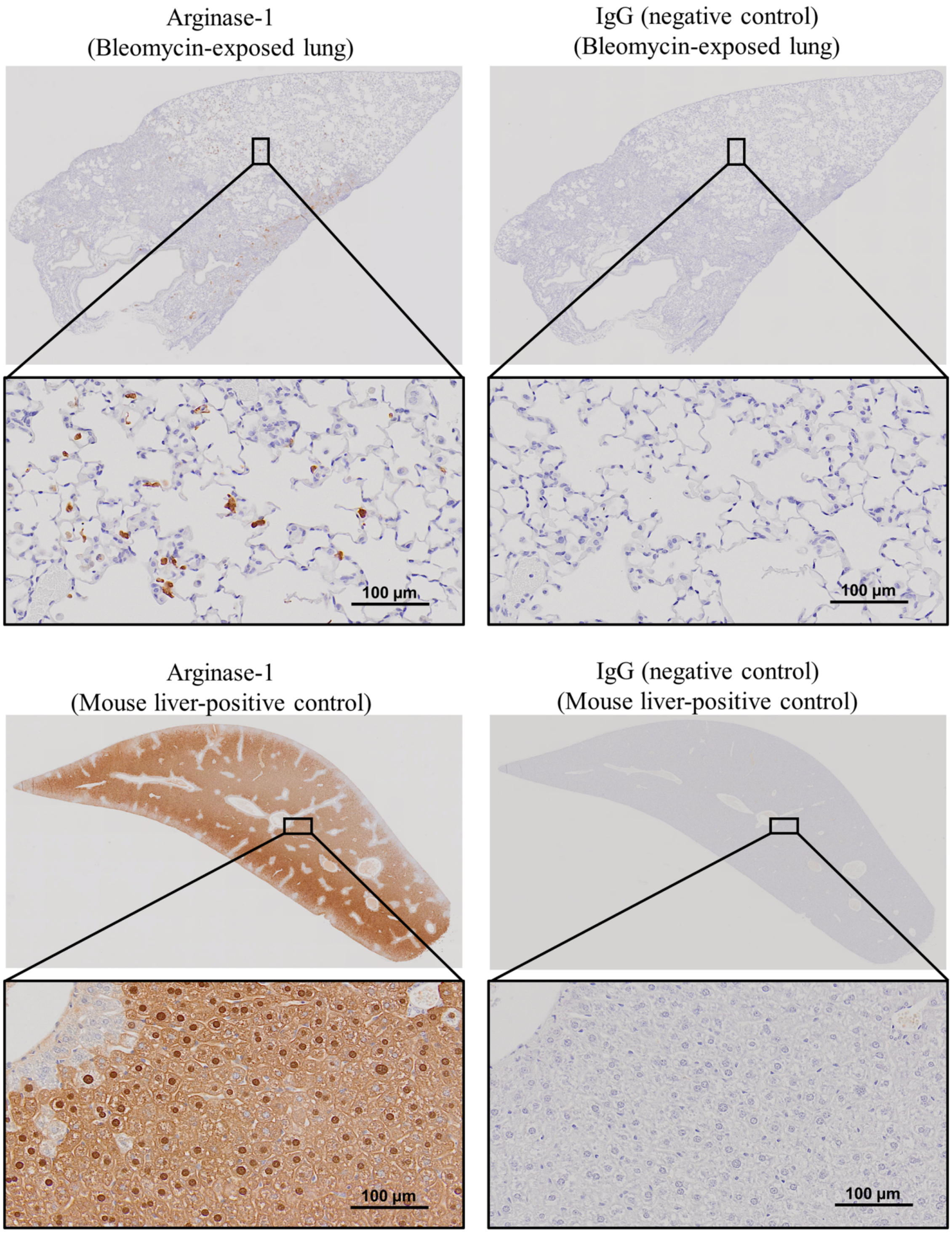

Supplementary Figure 2. Validation of mouse immunohistochemical staining of arginase-1 using positive and negative controls. Formalin-fixed sections of bleomycin-exposed lung tissues and control

liver tissues were stained with anti-arginase-1 antibody and a control (anti-IgG) antibody. Full tissue images and large scale inserts are shown.

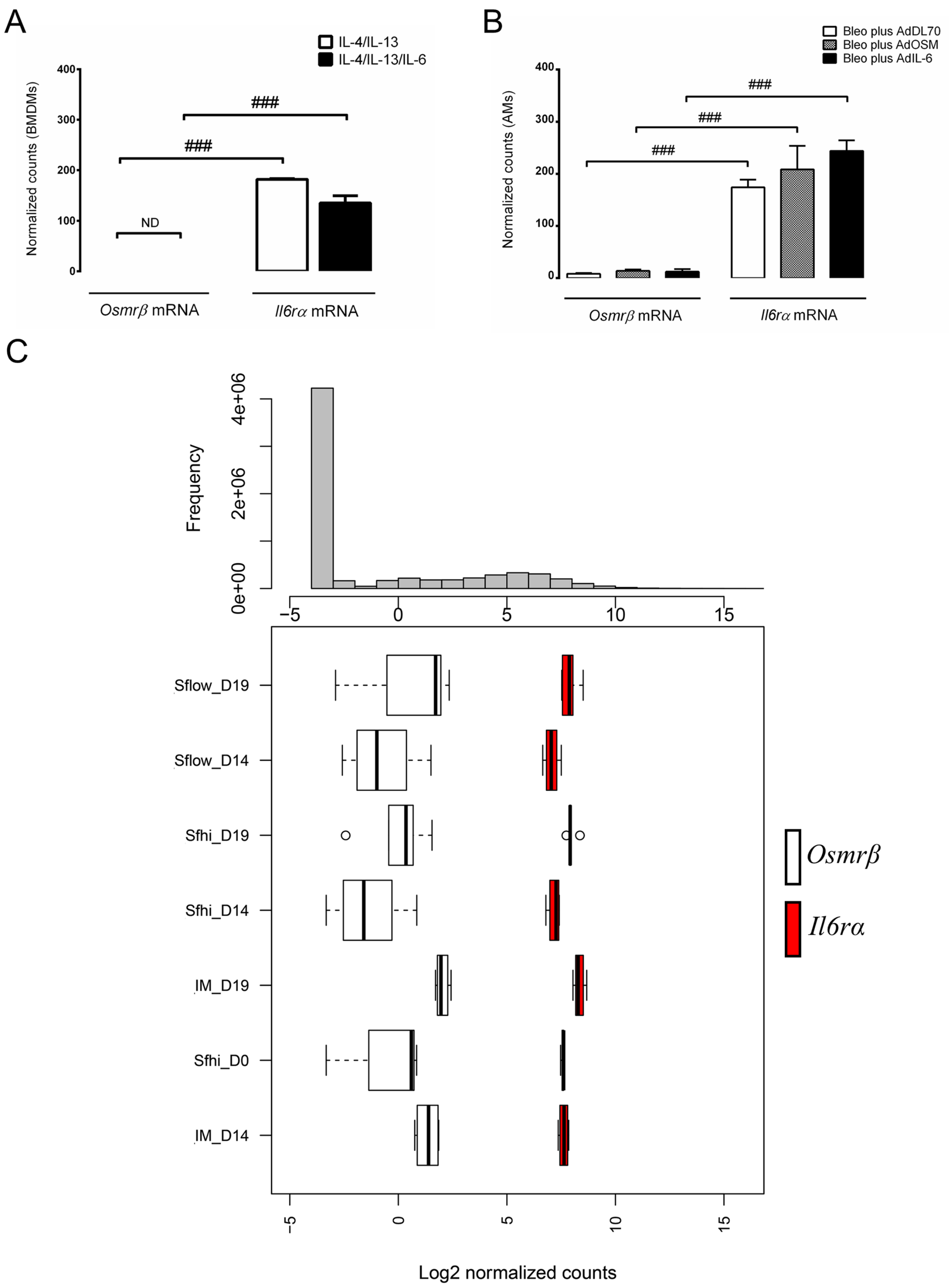

Supplementary Figure 3. Comparison of the expression of *Osmrβ* and *Il6rα* in BMDMs and lung macrophages. (A) *Osmrβ* and *Il6rα* mRNA expression from BMDMs exposed to IL-4/IL-13 and IL-4/IL-13/IL-6 for 30 hours. (B) *Osmrβ* and *Il6rα* mRNA expression from BALF AMs following 7 days of exposure to bleomycin plus AdDL70, AdOSM or AdIL-6. (C) Analysis of publically available GEO

database showing overall gene expression frequency profile as well as expression of *Osmrβ* (white-bars) and *Il6rα* (red-bars) in different macrophage populations within the lung. Sflow and Sfhi= Siglec F low and high alveolar macrophages, IM= interstitial macrophages, D0=non-bleomycin exposed lungs and D14/19= days of bleomycin exposure before sacrifice. Bar graphs represent mean ± SEM from 3-5 samples per group, ###:$P<0.001$ and significance was established using GraphPad, Prism 7.0 with one-way and two ANOVA using Newman-Keuls Multiple Comparison test. For Nanostring gene expression, lower than 5 counts was considered not detected "ND". Log2 transformed counts were analysed and plotted using R environment.

.

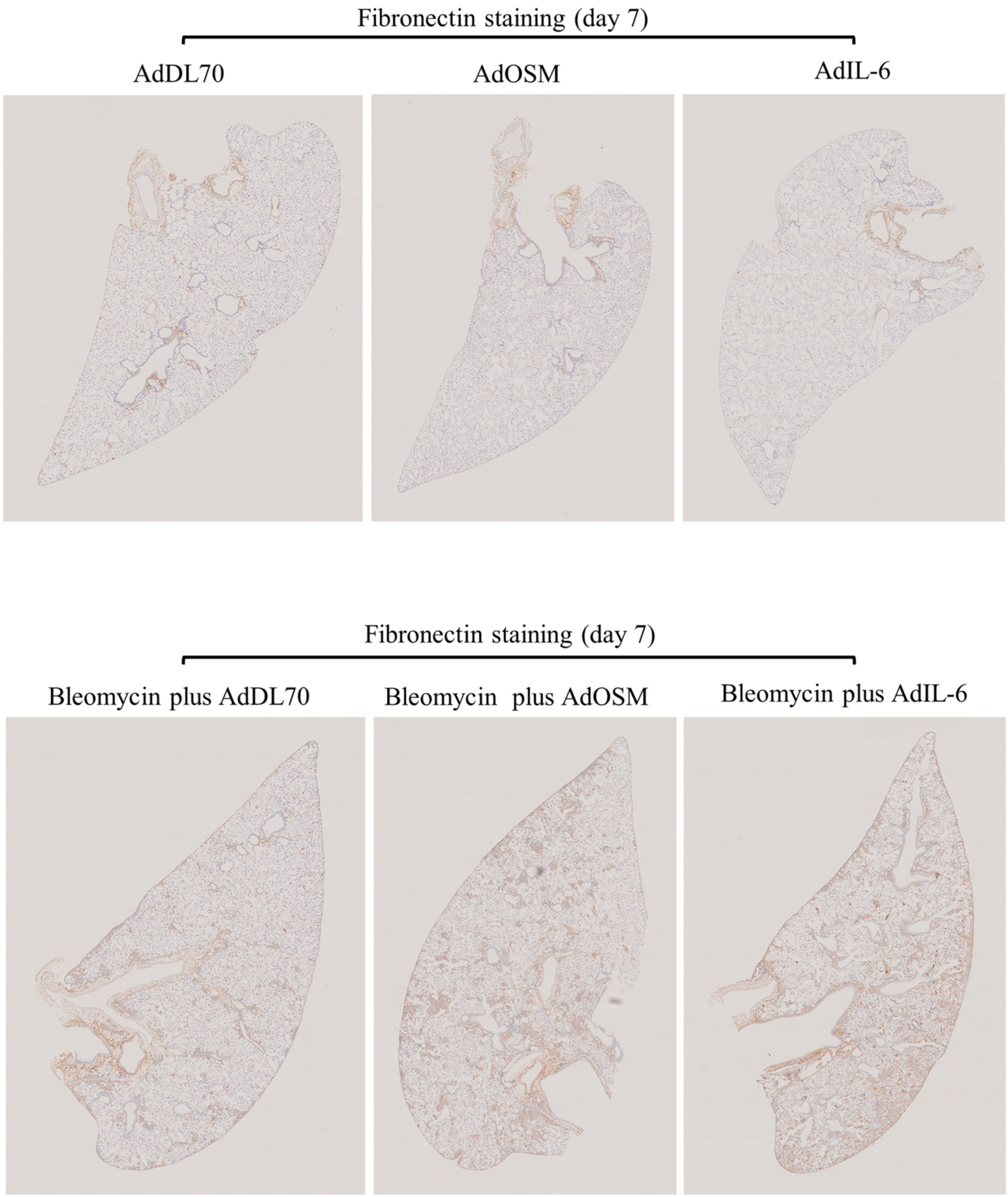

Supplementary Figure 4. Lung immunohistochemical staining of fibronectin in mice following in mice exposed to bleomycin plus AdOSM or AdIL-6 after 7 days of exposure of adenoviral vectors alone and with bleomycin.

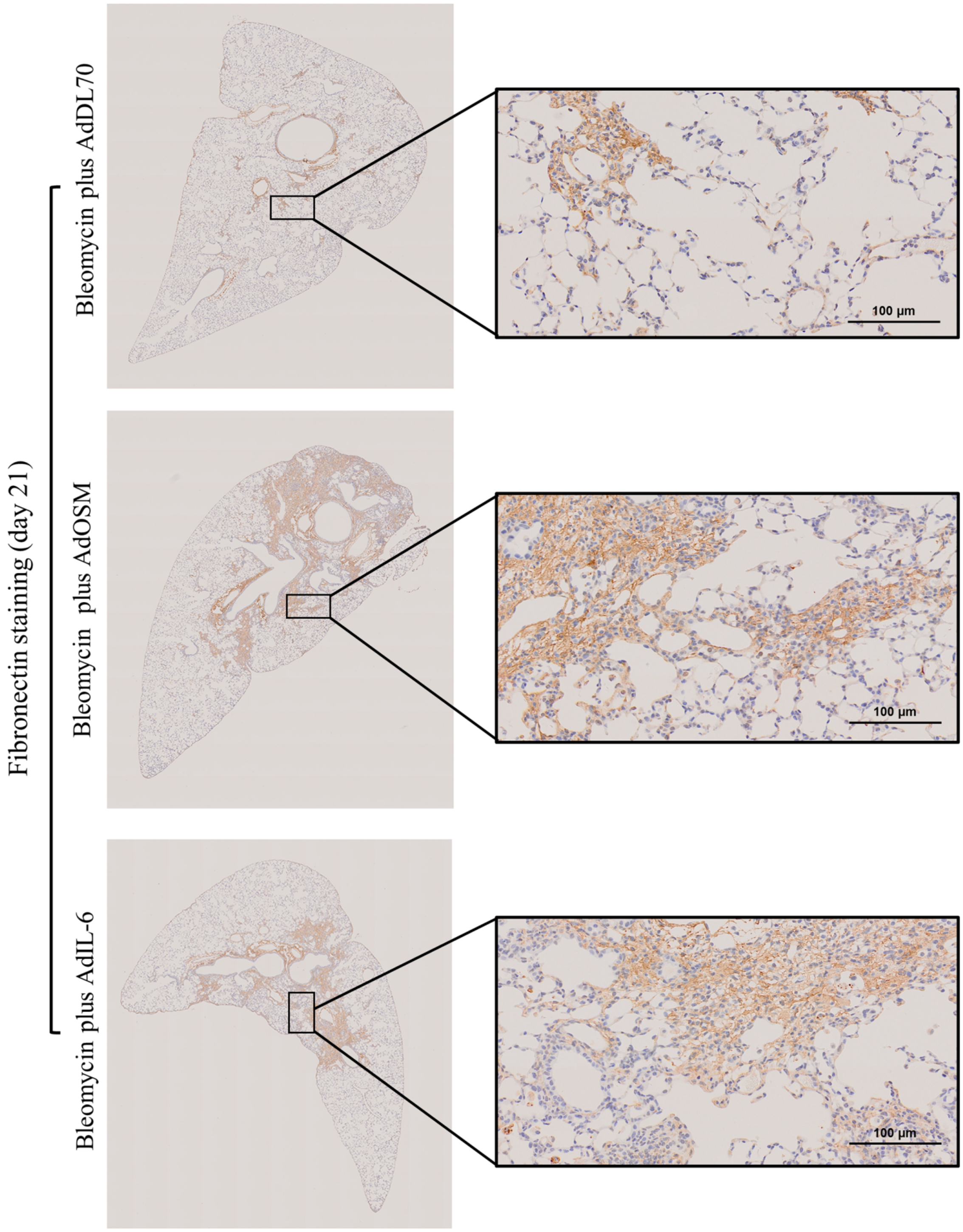

Supplementary Figure 5. Lung immunohistochemical staining of fibronectin in mice exposed to bleomycin plus AdOSM or AdIL-6 after 21 days of exposure.

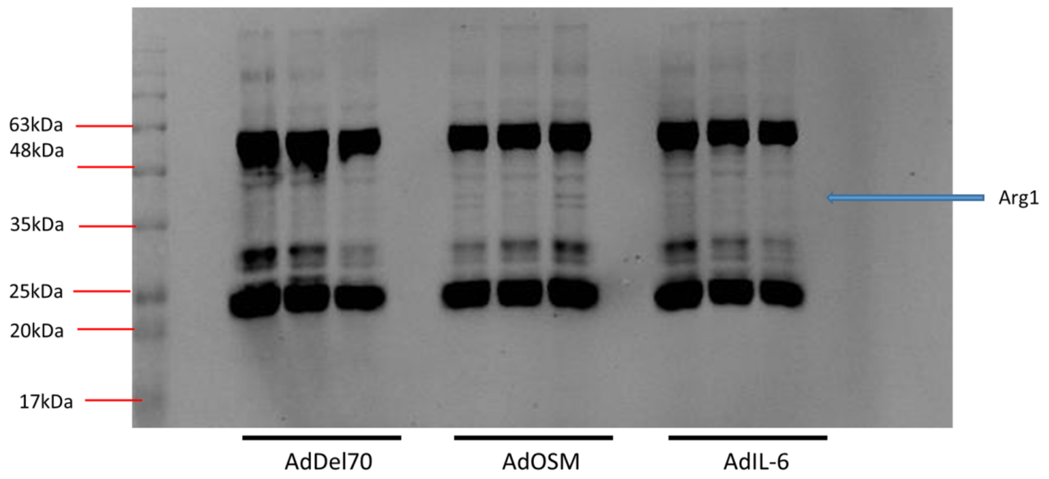

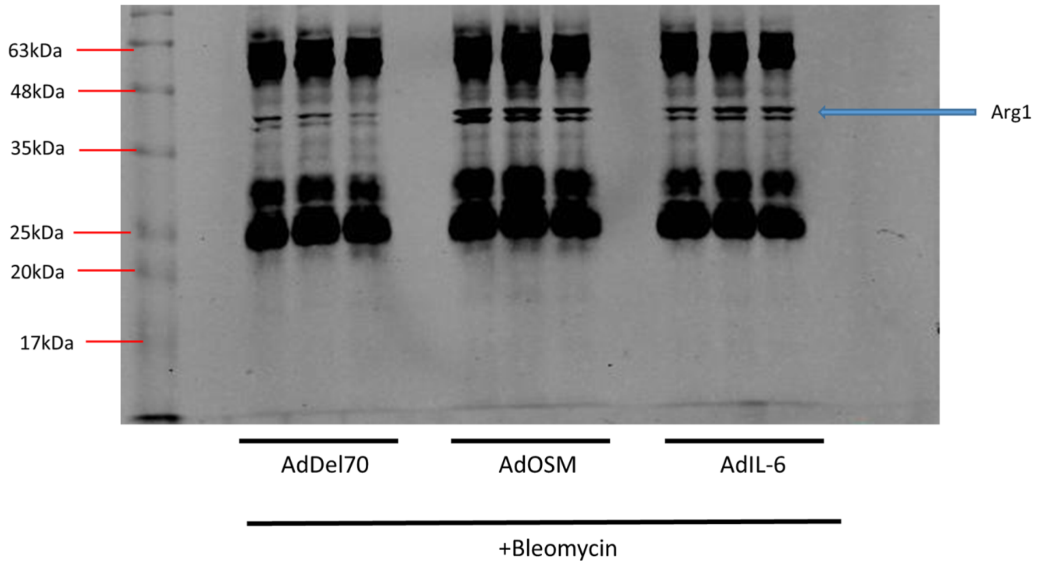

Supplementary Figure 6. Arginase-1 full length blots for figure 3A-3B

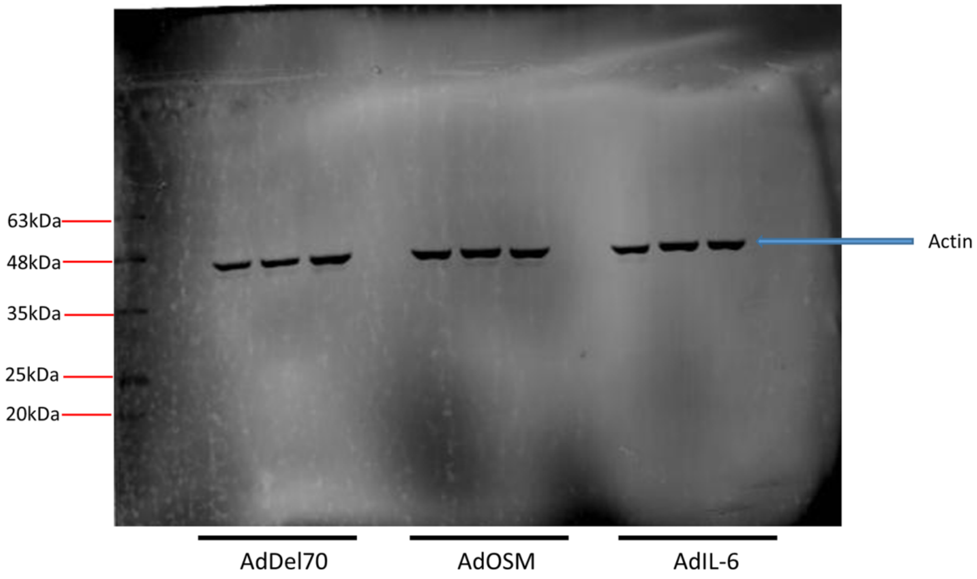

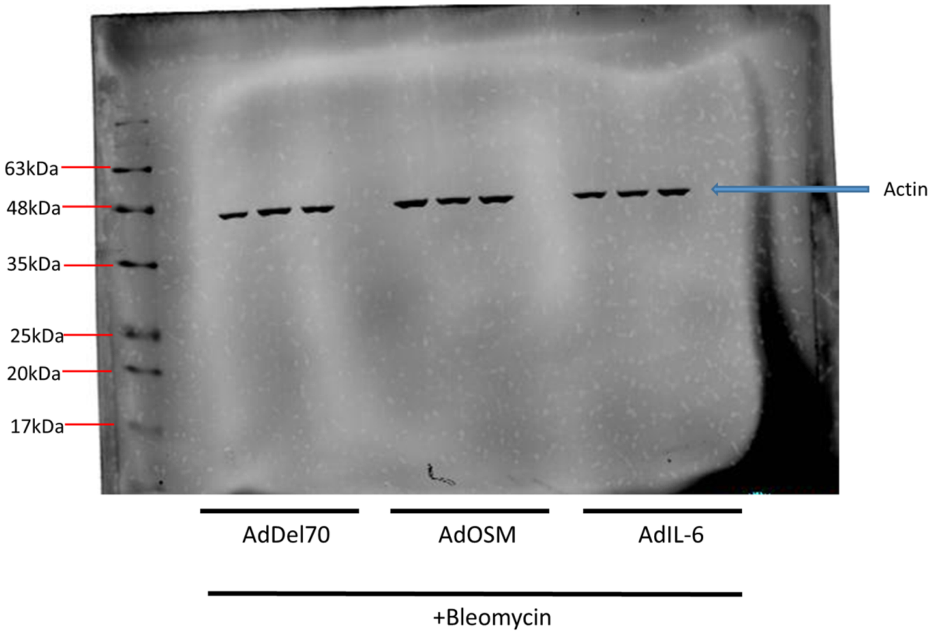

Supplementary Figure 7. Actin full length blots for figure 3A-3B

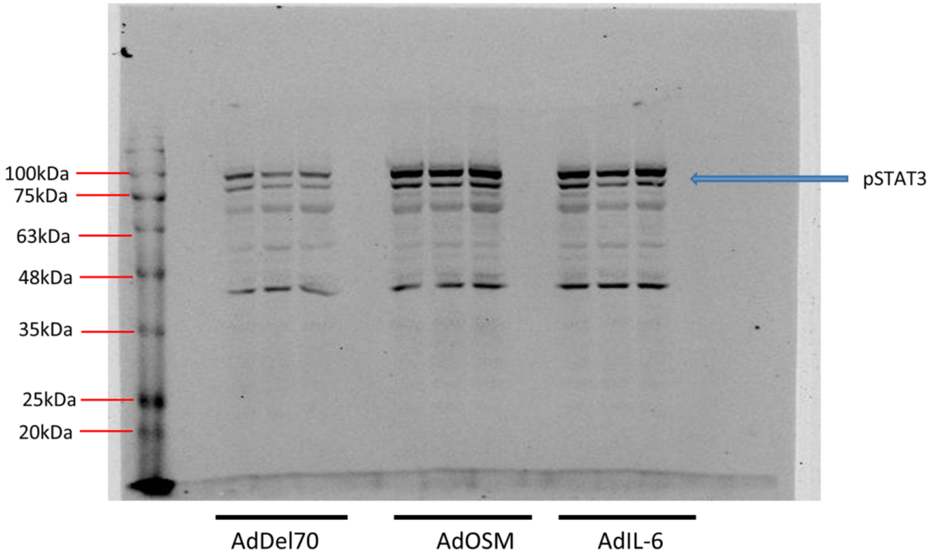

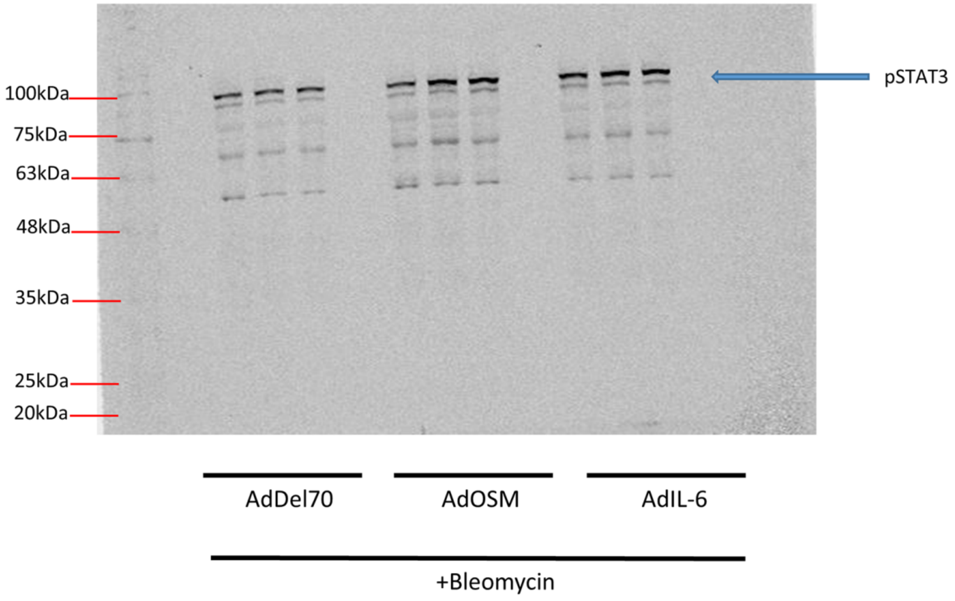

Supplementary Figure 8. pSTAT3 full length blots for figure 3A-3B

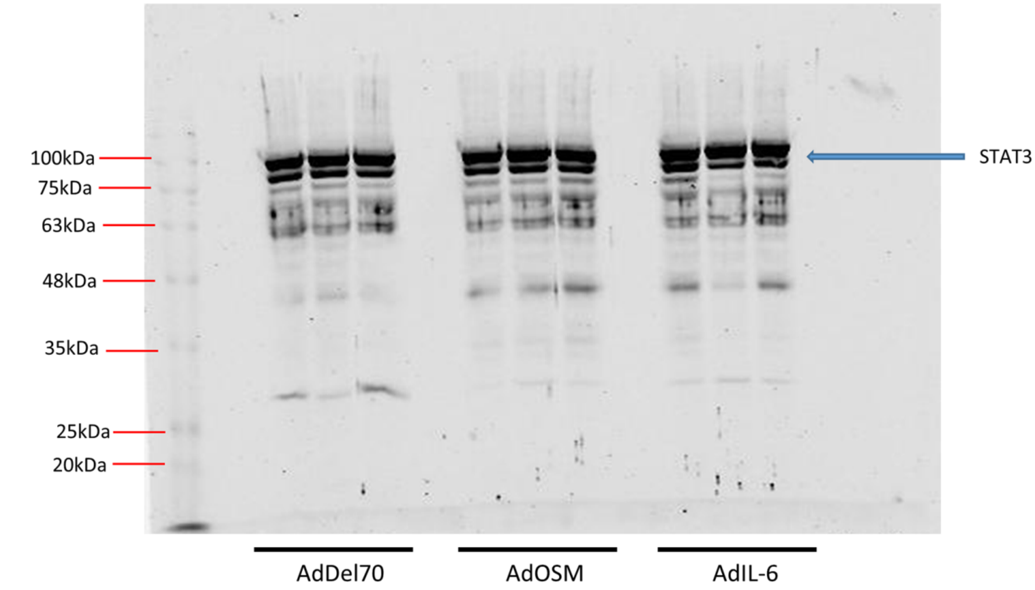

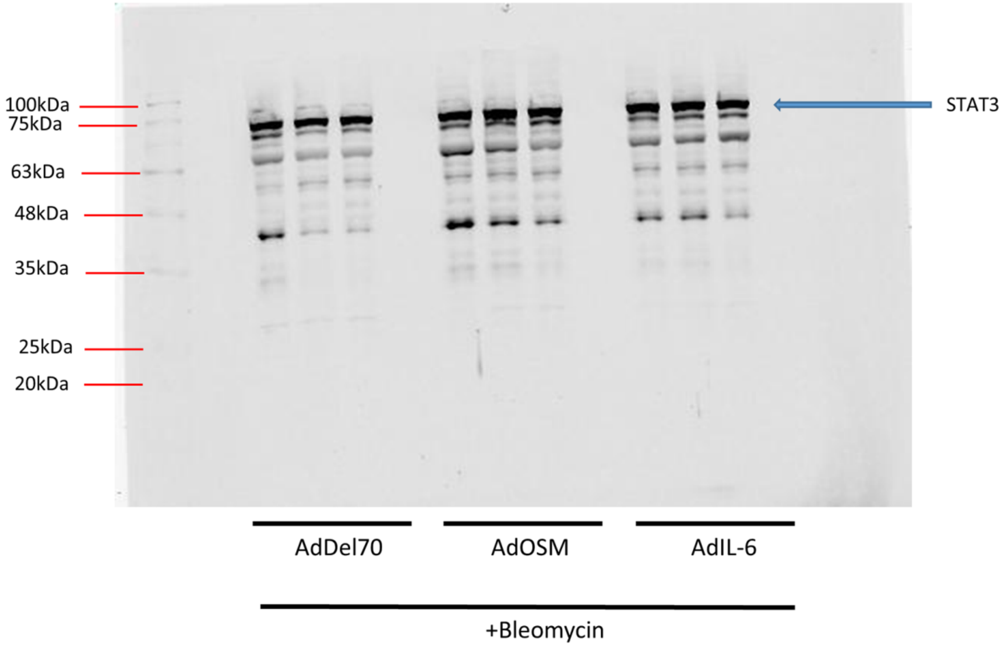

Supplementary Figure 9. STAT3 full length blots for figure 3A-3B

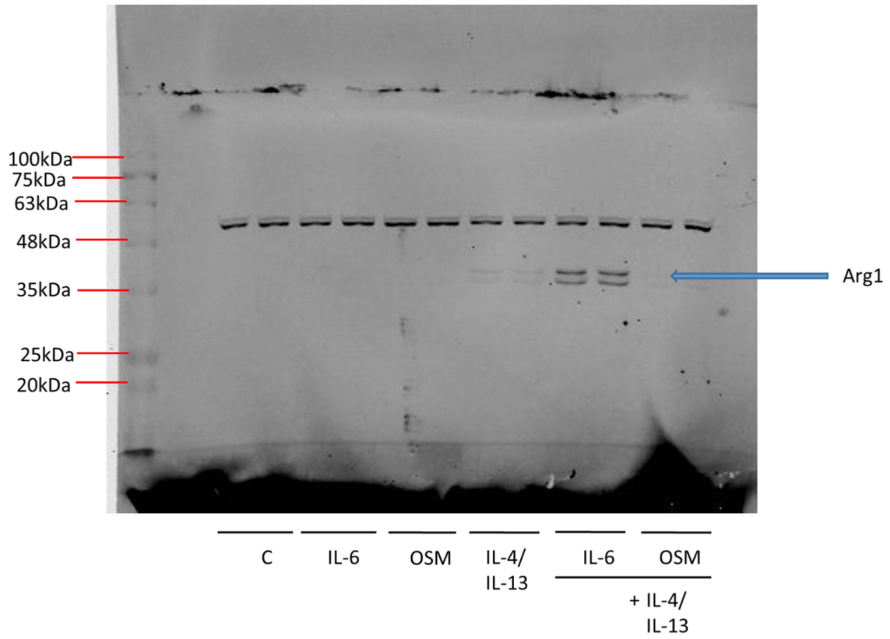

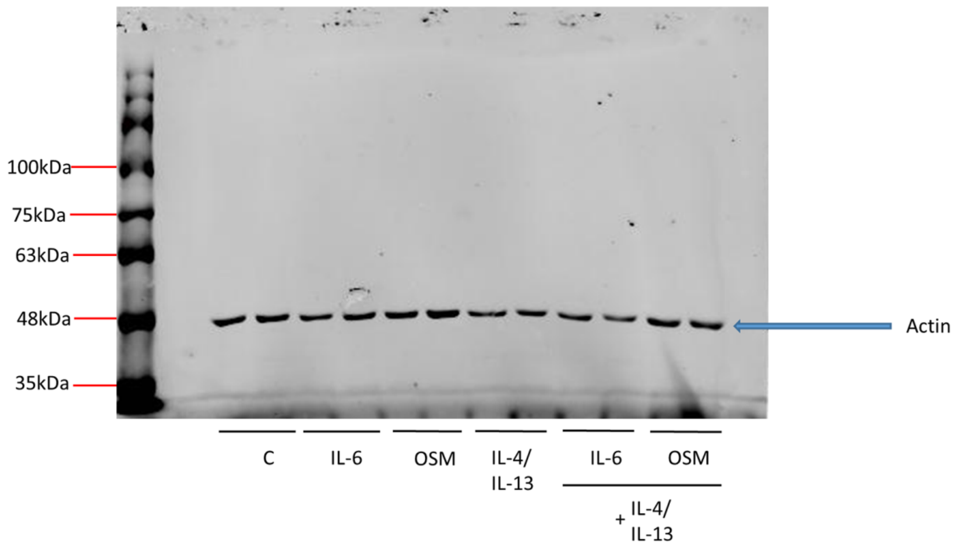

Supplementary Figure 10. Arginase-1 and Actin full length blots for figure 5E

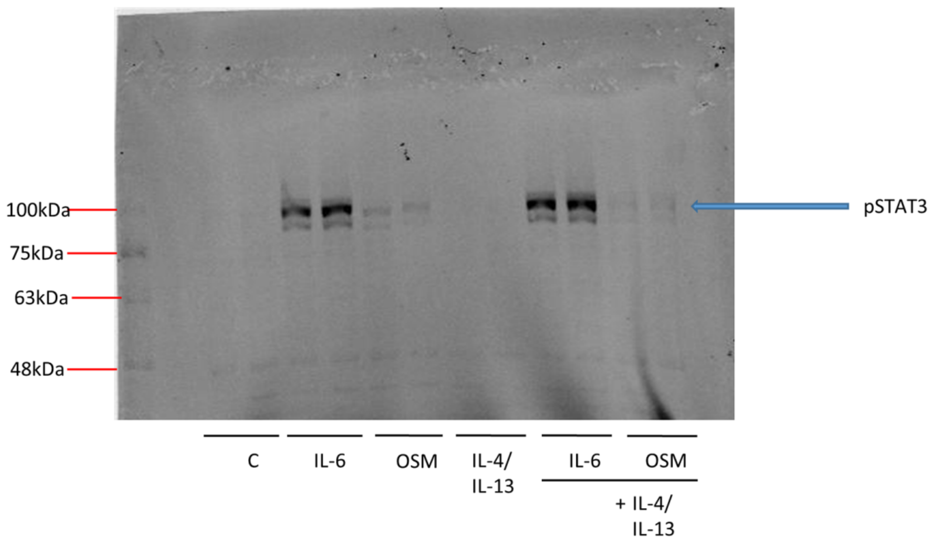

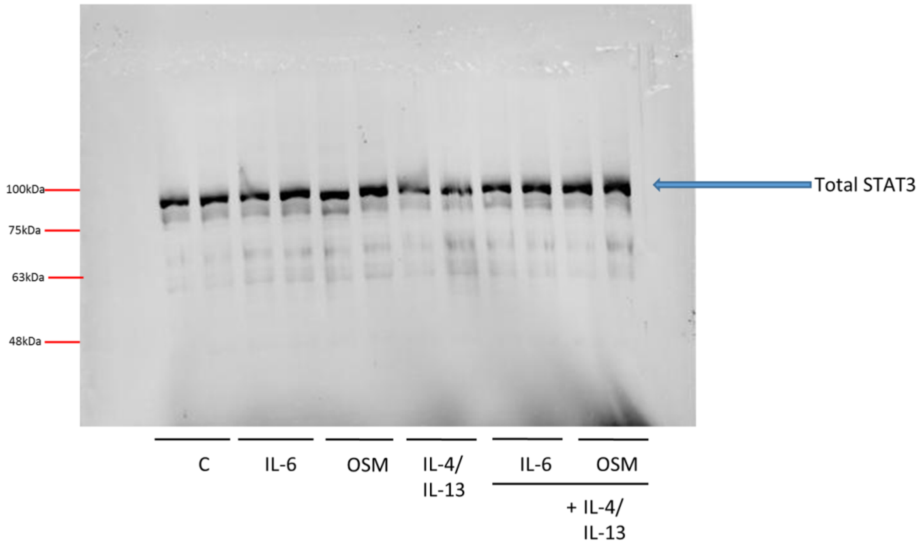

Supplementary Figure 11. pSTAT3 and STAT3 full length blots for figure 5E

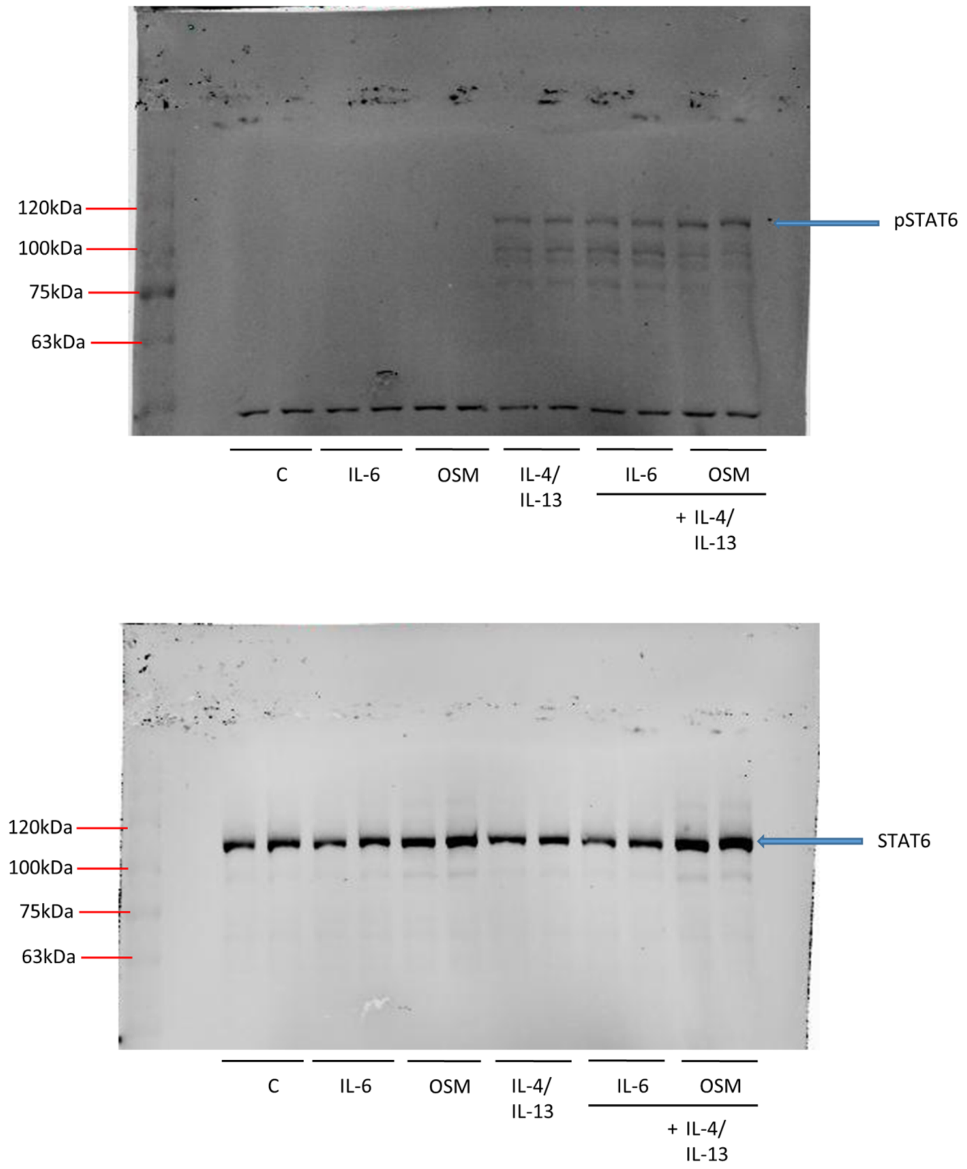

Supplementary Figure 12. pSTAT6 and STAT6 full length blots for figure 5E
